# Hierarchical Self‐Assembly of Capsule‐Shaped Zirconium Coordination Cages with Quaternary Structure

**DOI:** 10.1002/advs.202308445

**Published:** 2024-01-16

**Authors:** Shunfu Du, Shihao Sun, Zhanfeng Ju, Wenjing Wang, Kongzhao Su, Fenglei Qiu, Xuying Yu, Gang Xu, Daqiang Yuan

**Affiliations:** ^1^ State Key Laboratory of Structural Chemistry Fujian Provincial Key Laboratory of Materials and Techniques toward Hydrogen Energy Fujian Institute of Research on the Structure of Matter The Chinese Academy of Sciences Fuzhou Fujian 350108 P. R. China; ^2^ University of the Chinese Academy of Sciences Beijing 100049 P. R. China; ^3^ College of Chemistry Fuzhou University Fuzhou 350108 P. R. China

**Keywords:** hierarchical self‐assembly, hydrogen bonding, proton conductivity, quaternary structure, zirconium coordination cages

## Abstract

Biological macromolecules exhibit emergent functions through hierarchical self‐assembly, a concept that is extended to design artificial supramolecular assemblies. Here, the first example of breaking the common parallel arrangement of capsule‐shaped zirconium coordination cages is reported by constructing the hierarchical porous framework **ZrR‐1**. **ZrR‐1** adopts a quaternary structure resembling protein and contains 12‐connected chloride clusters, representing the highest connectivity for zirconium‐based cages reported thus far. Compared to the parallel framework **ZrR‐2**, **ZrR‐1** demonstrated enhanced stability in acidic aqueous solutions and a tenfold increase in BET surface area (879 m^2^ g^−1^). **ZrR‐1** also exhibits excellent proton conductivity, reaching 1.31 × 10^−2^ S·cm^−1^ at 353 K and 98% relative humidity, with a low activation energy of 0.143 eV. This finding provides insights into controlling the hierarchical self‐assembly of metal–organic cages to discover superstructures with emergent properties.

## Graphical abstract

The first example of breaking the common entropy‐dominated parallel arrangement of capsule‐shaped zirconium coordination cages is reported by constructing the hierarchical porous framework **ZrR‐1**. **ZrR‐1** adopts a quaternary structure resembling protein and contains 12‐connected chloride clusters, representing the highest connectivity for zirconium‐based cages reported thus far. **ZrR‐1** demonstrated unprecedented framework stability, high porosity, and superproton conductivity.

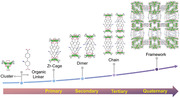

## 1 Introduction

In living organisms, hierarchical materials have evolved through natural selection, ensuring efficient substance and information transfer and exchange, such as the transfer of sodium and potassium ions across cell membranes.^[^ ^1^ ^]^ Inspired by biological systems, the controlled construction of hierarchical porous materials offers numerous possibilities for specific functions.^[^ ^2^ ^]^ However, artificial materials still lack the intelligence and functionality of proteins.^[^ ^3^ ^]^ To address this, one potential approach is to use complex cage‐like structures, known as cages, instead of simpler molecules, as building blocks due to their versatility and intricate arrangements.^[^ ^4^ ^]^ Among various cage types, metal–organic cages, with their unique 3D nature, precise stereochemistry, and complex topology, provide a powerful toolbox to create hierarchical porous materials that are inaccessible using conventional molecules and cages.^[^ ^5^ ^]^

The use of capsule‐shaped cages as synthons provides a simple, rapid, and convenient method to construct hierarchically porous materials. These capsules can be simplified as linear ligands that form the framework of various topological networks using reticular chemistry.^[^ ^6^ ^]^ However, previous investigations have shown that most capsule‐shaped cages tend to favor a parallel arrangement to increase the dominant translational entropy while decreasing the orientational entropy.^[^ ^7^ ^]^ To address this limitation, we propose to increase the hydrogen bond density to increase the energy of the system and thus direct the hierarchical self‐assembly of the cage toward higher‐order architectures. For this purpose, we have chosen a capsule‐shaped zirconium‐based cage with three outward *µ*‐OH groups, which can act as hydrogen bond donors. In addition, clusters of Cl^−^ ions with different geometry and connectivity (2‐c linear connectivity, 4‐c tetrahedral connectivity, and 8‐c cubic connectivity) act as hydrogen bond receptors.^[^ ^8^ ^]^ We envisage that non‐competitive solvents lacking strong H‐bond acceptors facilitate the formation of hydrogen bonds between *µ*‐OH and chloride ions, which contributes to the formation of more connected anion clusters, thus breaking the entropy‐dominated parallel arrangement and building hierarchical structures.^[^ ^9^ ^]^

**Scheme 1:**
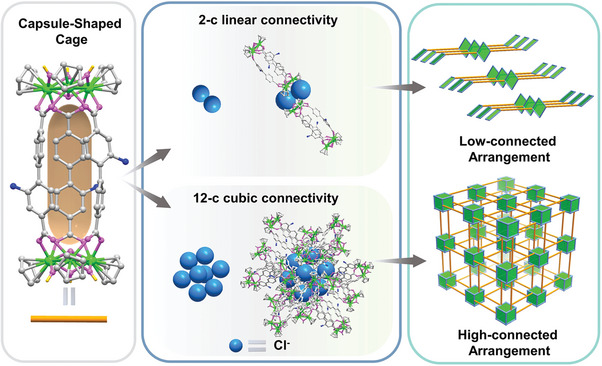
The self‐assembly of low‐connected and high‐connected superstructures based on capsule‐shaped zirconium cage as the building block.

Based on the above considerations, we achieved the highest 12‐c Cl^−^ clusters in Zr‐Cage‐based materials through directional induction assembly of non‐polar solvents. Moreover, breaking the common parallel arrangement of capsule‐shaped zirconium coordination cages not only constructed hierarchical order with well‐defined primary, secondary, tertiary, and quaternary structures, which we named **ZrR‐1**, but also realized the significant improvement in functionalities (**Scheme** **1**). Compared to the common parallel structure **ZrR‐2**, **ZrR‐1** showed unprecedented framework stability and porosity. In addition, **ZrR‐1** has a super proton conductivity of up to 1.31 × 10^−2^ S·cm^−1^ at 353 K and 98% relative humidity (RH). The activation energy (0.143 eV) obtained is among the lowest for cage‐based proton conductors, suggesting facile proton transfer.

## 2 Results and Discussion

**Figure 1:**
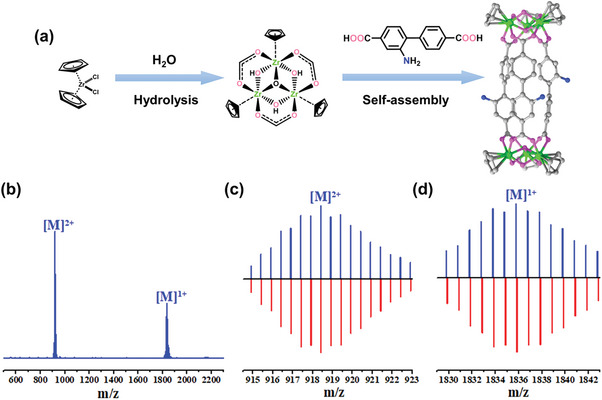
a) Self‐assembly of Zr‐Cage with H_2_L and ZrCp_2_Cl_2_. b) ESI‐TOF‐MS peaks of discrete Zr‐Cage. c) ESI‐MS of Zr‐Cage (2+) (blue) and simulated peaks (red). d) ESI‐MS of Zr‐Cage (1+) (blue) and simulated peaks (red).

In the past few years, we have achieved a major milestone by successfully synthesizing a series of Zr‐Cages and developing corresponding synthetic strategies in our subsequent studies.^[^ ^10^ ^]^ To prepare the capsule‐shaped cages, we used the linear ligand 2‐amino‐[1,1′‐biphenyl]−4,4′‐dicarboxylic acid (H_2_L) to assemble with ZrCp_2_Cl_2_ (**Figure** **1**a). The identity of **ZrR‐1** in solution was confirmed by high‐resolution electrospray ionization time‐of‐flight mass spectrometry (ESI‐TOF‐MS). We confirmed Zr‐Cage as the targeted capsule‐shaped assembly based on the measured isotopic peaks [M]^+^ (1835.81) and [M]^2+^ (918.41) (Figure 1b). These measurements were in excellent agreement with the simulated isotopic distributions 1835.82 and 918.4 (Figure 1c,d). To assess the chemical stability of Zr‐Cage, we conducted immersion tests in aqueous solutions at pH 3 and 9 were carried out, followed by analysis by ESI‐TOF‐MS. Importantly, the absence of significant changes in the MS spectra of Zr‐Cage indicated its remarkable stability over a wide pH range from 3.0 to 9.0 (Figure S1, Supporting Information).

Small structural changes, particularly in solvent molecules, can significantly influence the hierarchical self‐assembly process when using cages as building blocks. Strong polar solvents like *N*, *N*‐dimethylformamide, *N*, *N*‐dimethylacetamide, and methanol are commonly employed in Zr‐cage construction, and they often compete with chloride ions, resulting in low‐connected arrangements of capsule‐shaped cages through solvent molecule bridging. Such arrangements are not conducive to constructing hierarchical structures. On the other hand, noncompetitive solvents lacking strong H‐bond acceptors or donors can play a role in directional coordination bonding in extended crystalline frameworks.^[^ ^11^ ^]^

**Figure 2:**
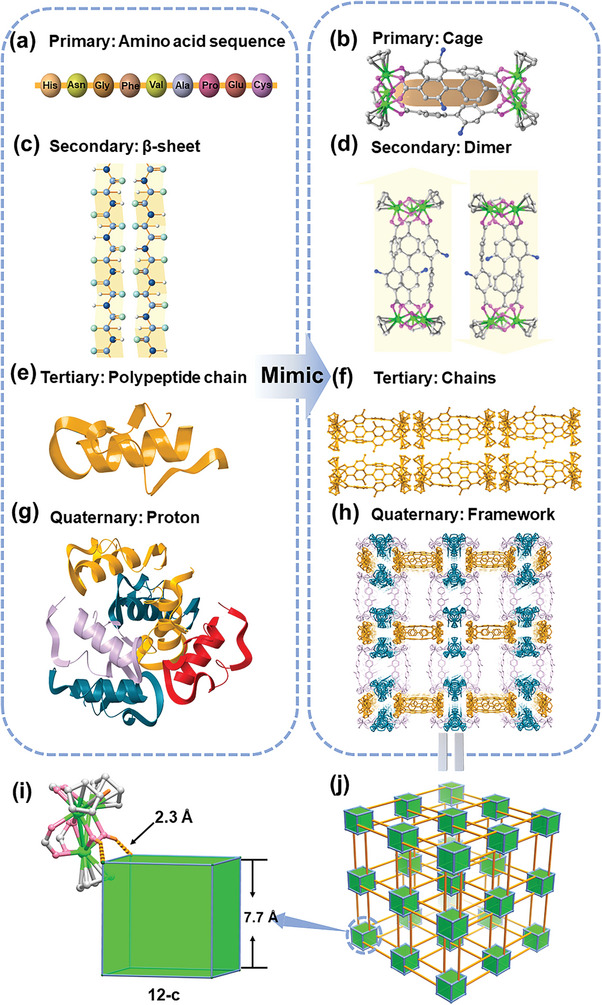
Hierarchical structure of proteins and **ZrR‐1**. Primary structure: amino acid sequence and capsule‐shaped cage (a) and (b). Secondary structure: β‐sheet and dimer (c) and (d). Tertiary structure: monomer and chains (e) and (f). Quaternary structure: proton and framework (g) and (h). (j) 3D topology structures. (i) Octahedral anionic cage. Atom labels: C, light grey; O, pink; N, blue; Zr, bright green; Cl, green.

In this study, we chose 1,4‐dioxane as a solvent to obtain a single crystal **ZrR‐1**. This cage crystallized in the cubic space group *P*m‐3. Analyzing the structure, we found that **ZrR‐1** is a supramolecular framework formed by the complex and delicate assembly of Zr‐cages. This led us to associate it with the hierarchical assembly process of proteins and to classify it into a well‐defined primary, secondary, tertiary, and quaternary structure to show its composition more clearly. In the primary structure of **ZrR‐1**, the cationic building block adopts a discrete capsule‐shaped geometry with V_2_E_3_ (V: vertex, E: edge) topology (**Figure** **2**b), and trinuclear zirconium clusters act as the vertices, while three bridging ligands form the edges. It is assembled by covalent and coordination bonds, which is like the amino acid sequence formed by covalent bonds. (Figure 2a). The amino groups point outward from the cavities, causing the two benzene rings in the ligand to be non‐coplanar. In addition, there is a cylindrical cavity exists in the inner pore with a height of 12.94 Å and a diameter of 2.68 Å. Two primary structures combine to form a supramolecular dimer (secondary structure) where one primary structure is oriented in the opposite direction to the other and the closest distance between them is 2.78 Å (Figure 2d). This is like the inverse parallel arrangement of the β‐sheet in a protein secondary structure (Figure 2c). As expected, the supramolecular dimer was identified by ESI‐TOF‐MS. The observed ion peaks at 1236.5492 correspond to [2M‐3Cl]^3+^ (Figure S2, Supporting Information). These dimers arrange axially to form a tertiary structure (Figure 2f), analogous to the polypeptide chain (Figure 2e). Finally, in the same way that peptide bonds interact with each other through hydrogen bonds to form the quaternary structure of a protein (Figure 2g), the linear tertiary structures of three axials are connected through hydrogen bonds at the nodes to form a quaternary structure with a large pore (Figure 2h; Figure S3, Supporting Information). This arrangement is unique because it is the only non‐parallel arrangement of a cage‐based material with a well‐defined quaternary structure among all reported M_2_L_3_ Zr‐cages (Table S2, Supporting Information).

During the hierarchical self‐assembly process of **ZrR‐1**, Cl^−^ ions form an octahedral anionic cage with a side length of 7.7 Å, resulting in a high order supramolecular framework. At each vertex of this cage, Cl^−^ forms hydrogen bonds with three OH groups from three Zr‐Cages in different directions, while two OH groups on each molecule together occupy one edge of the octahedron (Figures 2i; Figure S4, Supporting Information). This arrangement results in a 12‐connected cluster of Cl^−^ ions, which represents the highest number of connections among current Zr‐Cages. Despite having a moderate H···Cl distance of ≈2.3 Å and an O−H···Cl^−^ angle of ≈147.96°, the highly directional and dense hydrogen bonding significantly breaks the parallel arrangement, resulting in the formation of an exceptional hierarchical supramolecular porous network.

**Figure 3:**
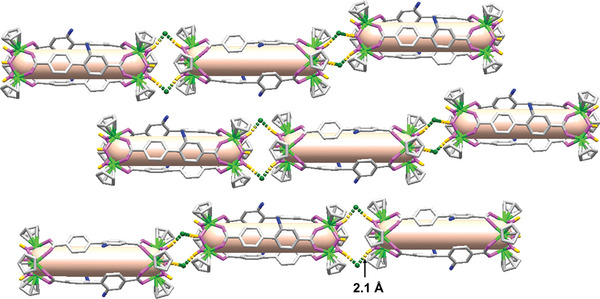
Parallel arrangement of Zr‐Cages in **ZrR‐2** and hydrogen bond connection.

When we attempted other methods, we could only achieve the common parallel arrangement of frameworks, **ZrR‐2** (**Figure** **3**; Figures S5 and S6, Supporting Information). In **ZrR‐2**, two OH groups from the zirconium cluster are bond to two adjacent vertices by two O−H···Cl^−^···H−O hydrogen bonds, with an H···Cl distance of ≈2.10 Å, and an angle of O−H···Cl^−^ ≈90.93°. It is evident that both the hydrogen bond strength and density in **ZrR‐2** are lower than those in **ZrR‐1**. To further analyze the inter‐cage hydrogen bonds in the frameworks, we calculated the Hirshfeld surface and 2D‐fingerprint plots using Crystal explorer software.^[^ ^12^ ^]^ In **ZrR‐1** and **ZrR‐2**, each Zr‐cage is connected to another 12 cages and 8 cages via bridging Cl^−^, respectively (Figures S7b and S8b, Supporting Information). The more regular shape of the Hirshfeld surface of **ZrR‐1** compared to **ZrR‐2** may be due to the higher symmetry packing mode of **ZrR‐1**(Figures S7a and S8a, Supporting Information). A noticeable peak corresponding to O−H··· Cl^−^ interaction is present in the fingerprint plot for both **ZrR‐1** and **ZrR‐2** (Figures S7c and S8, Supporting Informationc). In addition, the O−H···Cl^−^ contact contributes to 7.4% for **ZrR‐2**, while it increases to 9.7% for **ZrR‐1** (Figures S7d and S8d, Supporting Information). These results further confirm the higher hydrogen bond density in **ZrR‐1** compared to **ZrR‐2**, which contributes to its exceptional hierarchical supramolecular porous network.

**Figure 4:**
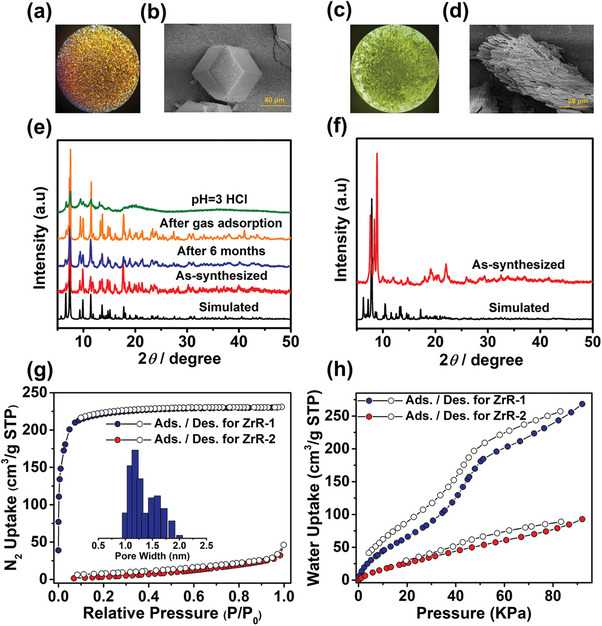
a,c) Optical images of **ZrR‐1** and **ZrR‐2** single crystals, respectively. b,d) SEM images of **ZrR‐1** and **ZrR‐2**. e) PXRD patterns of **ZrR‐1** in different state. f) PXRD patterns of **ZrR‐2**. g) N_2_ sorption isotherm for **ZrR‐1** and **ZrR‐2** at 77 K. Inset shows pore size distribution for **ZrR‐1**. h) Water sorption isotherm for **ZrR‐1** and **ZrR‐2** at 273 K.

**ZrR‐1** and **ZrR‐2**, although sharing the same building blocks, exhibit significant differences in their structures and properties due to their different modes of assembly. The most striking difference is in their morphology; **ZrR‐1** forms brown cubic crystals (**Figure** **4**a,b), while **ZrR‐2** forms yellow parallelepiped crystals (Figure 4c,d). Furthermore, TEM images also show significant morphological disparities between **ZrR‐1** and **ZrR‐2** (Figure S9, Supporting Information). Stability is another prominent difference between the two. Typically, cage‐based frameworks are unstable, especially when removed from the mother liquor, as observed in the case of **ZrR‐2** (Figure 4f). However, **ZrR‐1** maintains its stability even after gas adsorption experiments, exposure to a humid environment for six months, or immersion in an acidic solution for 24 h. Its PXRD patterns remain almost unchanged (Figure 4e), and it exhibits remarkable thermal stability, with PXRD patterns remaining largely unchanged over a temperature range of 30–100 °C (Figure S10, Supporting Information). Additionally, its crystallinity allows diffraction spots to be obtained even after gas adsorption tests, which is unusual for supramolecular frameworks based on metal–organic cages (Figures S11 and S12, Supporting Information). The N_2_ adsorption performance provides insight into the stability and porosity of a structure. **ZrR‐1** shows excellent N_2_ adsorption, exhibiting a fully reversible type I isotherm with a BET surface area of 879 m^2^ g^−1^. The pore size distribution of **ZrR‐1**, as determined by the Horvath–Kawazoe (H‐K) method, is centered at 1.2 and 1.7 nm (Figure 4g). **ZrR‐1** also shows significant adsorption of CO_2_, CH_4_, C_2_H_n_, and C_3_H_n_ hydrocarbons (Figures S13–S15, Supporting Information). On the other hand, **ZrR‐2** has almost no N_2_ adsorption. There is also a significant disparity between **ZrR‐1** and **ZrR‐2** concerning water adsorption, with **ZrR‐1** showing an adsorption capacity almost three times higher than that of **ZrR‐2** (Figure 4h). Overall, the high density of hydrogen bonds in this novel assembly ensures that the material has excellent framework stability, endowing it with outstanding adsorption properties. These differences in structures and properties between **ZrR‐1** and **ZrR‐2** highlight the importance of assembly methods in determining the properties of hierarchical supramolecular porous networks based on metal–organic cages.

**Figure 5:**
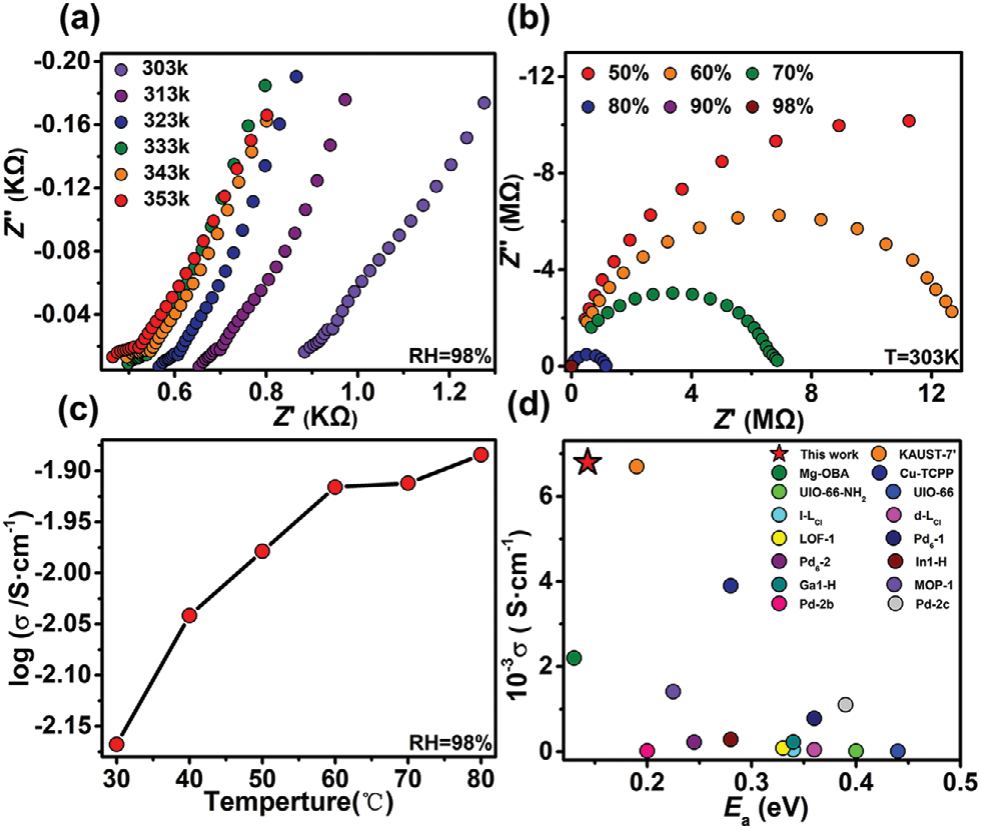
a) Temperature dependence of Nyquist plots for **ZrR‐1** at 98% RH. b) RH dependence of Nyquist plots for **ZrR‐1** at 303 K. c) Temperture‐dependent proton conductivity of **ZrR‐1** at 98% RH. d) Comparison of proton conductivity and *E*_a_ with some of reported Cages and MOFs.

The hierarchical structure within an organism often facilitates specific functions, among which the physiological process of charge transfer across the cell membrane holds significant importance. Therefore, we measured the proton conductivity (*σ*, S cm^−1^) of pelletized **ZrR‐1** samples using an alternating‐current (A.C.) impedance spectrometer at different humidity and temperature conditions. At ambient temperature (303 K) and 98% RH, **ZrR‐1** is an excellent conductor with the values reaching up to 6.80 × 10^−3^ S cm^−1^. It is noteworthy that the value of 6.80 × 10^−3^ S cm^−1^ at 98% RH reported here is not only the highest value yet recorded for a hydrated cage sample but is also comparable to the proton conductivity of an acid‐impregnated MOF material (Tables S5 and S6, Supporting Information).^[^ ^13^ ^]^ The corresponding conductivity values increase from 6.80 × 10^−3^ to 1.05×10^−2^ S cm^−1^ upon heating from 303 to 323 K. Although the values of proton conductivity have been steadily growing in cages and MOFs, there are a handful of materials that exceed the benchmark of 10^−2^ S cm^−1^ relatively mild temperatures.^[^ ^14^ ^]^ Raise the temperature, and the conductivity value is further increased to 1.31 × 10^−2^ S cm^−1^ at 353 K (**Figure** **5**a,c; Table S4, Supporting Information).

To further investigate whether water plays a role in the proton transport mechanism, impedance measurements were performed at 303 K in the range of 50% to 98% RH. The results demonstrated a significant dependence of the conductivity of **ZrR‐1** on humidity. At 303 K and 50% RH, **ZrR‐1** exhibited a low proton conductivity of 2.58 × 10^−7^ S cm^−1^. However, as the humidity increased to 98% RH, its proton conductivity substantially improved to 6.80 × 10^−3^ S cm^−1^ (Figure 5b: Figures S16 and S17, and Table S3, Supporting Information). This difference indicates that the 3D pore in **ZrR‐1** can accommodate more water molecules, which may form a hydrogen bonding network, facilitating proton conduction. Based on the Arrhenius plot, the calculated activation energy (*E*_a_) for proton transfer in **ZrR‐1** is 0.143 eV (Figure S18, Supporting Information). This value is the lowest among currently known cages with proton conductivity (Table S5, Supporting Information), and it is also lower than most MOF‐based materials. This suggests that the Grotthuss mechanism (*E*_a_ <0.4 eV) contributes predominantly to the proton conduction. In **ZrR‐1**, The framework does not contain common hydrophilic functional groups (‐SO_3_H, ‐PO_3_H, ‐COOH) that are typically used to enhance proton conductivity, and no active proton source is introduced into the pore by synthetic post‐treatment/modification.^[^ ^15^ ^]^ Under both conditions, the proton conductivity and *E*_a_ are superior to most similar MOF and cage materials. (Temperature range: 298–303 K, RH: 95% to 98%). Moreover, PXRD patterns demonstrated that **ZrR‐1** could maintain its structural integrity even after proton‐conducting experiments (Figure S19, Supporting Information), suggesting its potential application in proton‐conducting systems. These findings highlight the promising prospects of **ZrR‐1** as a material for proton conduction and its potential utility in related applications.

## 3 Conclusion

In conclusion, our study has achieved the successful construction of a supramolecular framework with a well‐defined quaternary structure. This breakthrough was achieved by disrupting the typical parallel arrangement of capsule‐shaped zirconium coordination cages through a controlled synthesis strategy. The resulting unusual assembly pattern led to significant improvements in stability, BET surface area, and proton conductivity. Our findings demonstrate that controlled hierarchical self‐assembly of capsule‐shaped zirconium coordination cages offers a promising molecular platform for exploring hierarchical superstructures that go beyond the capabilities of traditional self‐assembled building blocks and simple cages. This advancement paves the way for developing novel materials with exceptional properties. Overall, the discovery of this hierarchical supramolecular porous network showcases the potential for designing advanced materials using sophisticated assembly techniques, which can open new avenues for exploring and utilizing emergent materials in various applications.

## Conflict of Interest

The authors declare no conflict of interest.

## Supporting information

Supporting Information

Supporting Information

## Data Availability

The data that support the findings of this study are available in the supplementary material of this article.
