# Supplementary material for: Hierarchical Self‐Assembly of Capsule‐Shaped Zirconium Coordination Cages with Quaternary Structure
Source: Adv Sci (Weinh). 2024 Jan 16;11(11):2308445. doi: 10.1002/advs.202308445 (PMC10953209; doi:10.1002/advs.202308445)
Supplement: Supplementary file 1 — Supporting Information [file ADVS-11-2308445-s001.pdf]

ADVANCED SCIENCE
Open Access

Supporting Information

for *Adv. Sci.*, DOI 10.1002/advs.202308445

Hierarchical Self-Assembly of Capsule-Shaped Zirconium Coordination Cages with Quaternary Structure

Shunfu Du, Shihao Sun, Zhanfeng Ju, Wenjing Wang, Kongzhao Su, Fenglei Qiu, Xuying Yu, Gang Xu and Daqiang Yuan**

Supporting Information
©Wiley-VCH 2021
69451 Weinheim, Germany

Hierarchical Self-Assembly of Capsule-Shaped Zirconium Coordination Cages with Quaternary Structure

Shun-Fu Du,[a] [b] Shi-Hao Sun,[a] Zhan-Feng Ju,[a] [b] Wen-Jing Wang,[a] [b] Kong-Zhao Su,[a] [b] Feng-Lei Qiu,[a] [c] Xu-Ying Yu,[a] [b] Gang Xu,*[a] [b] and Da-Qiang Yuan*[a] [b]

Section S1. General Procedures, Materials, and Instrumentation

Zirconocene dichloride, *N*,*N*-Dimethylformamide (DMF), 1, 4-dioxane, and 2-amino-[1,1'-biphenyl]-4,4'-dicarboxylic acid were commercially available and used without further purification. Elemental analyses for C, H, and N were performed on an Elementar Vario MICRO elemental analyzer. Fourier transform infrared (FT-IR) spectra of the samples were recorded on KBr pellets in the 4000-400 cm^{-1} range using a PerkinElmer Spectrum One FT-IR spectrometer. Gas sorption data were obtained on Micromeritics ASAP2020. Single-crystal X-ray data were collected on an XtaLAB Synergy R, HyPix four-circle diffractometer with PhotonJet R (Cu) X-ray Source. The proton conductivity studies
The conductivity of **ZrR-1** was calculated by the equation:
$\sigma = L / RS$ (1)
Where σ represents conductivity, *L* is the thickness, *S* is the area of the measured plate, and *R*(Ω) is the resistance measured from the Nyquist plot.
Their activation energy (E_a) was calculated by the data of conductivity in the range of 30 to 80 °C at 98% RH with an Arrhenius equation:
$\ln(T) = \ln A - E_a / (k_B T)$ (2)
In the above equation, k_B represents the Boltzmann constant, and A is the pre-exponential factor.

Section S2. Synthesis of complexes

ZrR-1. Cp_2ZrCl_2 (0.015 g) and 2-amino-[1,1'-biphenyl]-4,4'-dicarboxylic acid (0.005 g) were dissolved in a mixture of 1 mL 1,4-Dioxane and 3 drops of distilled water, and the mixture was then heated at 65 °C for 12 h. After slowly cooling down to room temperature, brown block crystals were obtained. IR (KBr pellet, cm^{-1}): 3401, 1608, 1575, 1526, 1419, 1186, 1151, 1110, 1076, 1018, 819, 775, 727, 671, 619, 547, 472. Elemental analysis (%): calc. for **ZrR-1**·1,4-Dioxane·15H_2O ($Zr_6C_{76}O_{37}N_3H_{101}Cl_2$): C 40.27, H 4.49, N 1.85 Found: C 40.45, H 4.60, N 1.82.
ZrR-2. Cp_2ZrCl_2 (0.025 g) and 2-amino-[1,1'-biphenyl]-4,4'-dicarboxylic acid (0.008 g) were dissolved in a mixture of 1 mL *N*,*N*-Dimethylformamide and 3 drops of distilled water, and the mixture was then heated at 65 °C for 12 h. After slowly cooling down to room temperature, yellow block crystals were obtained. IR (KBr pellet, cm^{-1}): 3624, 3365, 1660, 1606, 1573, 1516, 1411, 1244, 1186, 1149, 1103, 1064, 1018, 952, 869, 813, 773, 727, 671, 613, 476. Elemental analysis (%): calc. for **ZrR-2**·7DMF·5H_2O ($Zr_6C_{93}O_{32}N_{10}H_{122}Cl_2$): C 44.50, H 4.90, N 5.58 Found: C 44.47, H 5.07, N 5.1.

Section S3. Single Crystal X-ray Diffraction Analyses

Single-crystal X-ray data of **ZrR-1** and **ZrR-2** are collected on an XtaLAB Synergy R, HyPix four-circle diffractometer with PhotonJet R (Cu) X-ray Source. The structures were solved by direct methods and refined by full-matrix least-squares on F^2 with anisotropic displacement using the *SHELXTL* software package. The non-H atoms were treated anisotropically, whereas the aromatic and hydroxyl- and alkyl-hydrogen atoms were placed in calculated, ideal positions and refined as riding on their respective carbon atoms. In these structures, free solvent molecules were highly disordered, and attempts to locate and refine the solvent peaks were unsuccessful. The diffused electron densities resulting from these residual solvent molecules were removed from the data set using the *SQUEEZE* routine of *PLATON* and refined further using the data generated. The contents of the solvent region are not represented in the unit cell contents in the crystal data. The data collection and refinement details are included in the CIF file in the supporting information. The details for data collection and refinement are listed in Table S1. Crystallographic data for the structures reported in this paper have been deposited in the Cambridge Crystallographic Data Center with CCDC Number: 2284939-2284940 for **ZrR-1** and **ZrR-2**.

Section S4. Additional Figures and Tables

Table S1. Crystal data and structure refinement for **ZrR-1** and **ZrR-2**.

	**ZrR-1**	**ZrR-2**
Formula	$C_{72}H_{62}Cl_2N_3O_{20}Zr_6$	$C_{81}H_{84}Cl_2N_6O_{23}Zr_6$
*F*w	1907.46	2127.76
Crystal system	cubic	triclinic
Space group	*P*m-3	*P*-1
a, Å	26.8894(2)	15.1238(2)
b, Å	26.8894(2)	15.5214(2)
c, Å	26.8894(2)	25.2844(3)
α, °	90	98.1180(10)
β, °	90	95.6860(10)
γ, °	90	112.6250(10)
V, $Å^3$	19442.1(4)	5346.69(12)
Z	6	2
Dc, g/cm^3	0.977	1.322
μ, mm^{-1}	4.549	5.598
Reflns collcted	25851	75012
2θ range, °	3.287 to 76.890	3.146 to 76.854
F (000)	5694	2140
GOF on F^2	1.034	1.031
R_1/wR_2 (I>2σ(I))	R_1 = 0.0596<br>wR_2 = 0.1805	R_1 = 0.0655<br>wR_2 = 0.1716
R_1/wR_2 (all data)	R_1 = 0.0750<br>wR_2 = 0.1994	R_1 = 0.0811<br>wR_2 = 0.1882

Table S2. **Crystallographic data of capsule-shaped Zr-cage have been reported.**

Cage	Space group	a (Å)	b (Å)	c (Å)	α (°)	β (°)	γ (°)	refs
Zr-MC-1	P-1	14.799	16.2691	17.0541	72.965	80.879	80.069	[1]
Zr-MC-2	P-1	10.7932	23.6272	25.0116	67.089	78.072	77.205	[1]
Zr-MC-3	P21/c	39.583	11.3619	19.236	90	91.681	90	[1]
Zr-MOC-2	C2/c	20.042	17.763	26.962	90	106.975	90	[2]
MOP-1	R-3c	16.2679	16.2679	84.1291	90	90	120	[3]
ImBDC-Zr	P63/m	16.2827	16.2827	17.437	90	90	120	[4]

ImBPDC-Zr	Cmcm	27.4283	19.573	18.1855	90	90	90	[4]
ImTPDC-Zr	P-1	15.3527	21.4377	26.732	68.372	83.209	69.958	[4]
UMC-1	P21	13.093	20.8	39.643	90	94.91	90	[5]
UMC-2	P21/n	31.681	13.796	51.247	90	100.14	90	[5]
NUT-101	C2/c	44.23	23.295	10.952	90	93.116	90	[6]
SCC-2	P21/c	26.2835	24.1855	35.3251	90	90.592	90	[7]
SCC-3	C2/c	40.826	31.107	26.162	90	103.758	90	[7]
SCC-6	C2/c	40.982	23.6712	33.668	90	107.606	90	[7]
Compound-1	$P2_12_12$	19.8311	20.8521	18.2738	90	90	90	[8]
Compound-2	Pccn	22.0951	20.0854	27.591	90	90	90	[8]
Compound-3	$P6_3/m$	21.7333	21.7333	30.7272	90	90	120	[8]
1C	$P2_1/c$	15.0641	23.6781	21.9714	90	96.323	90	[9]
2C OTf	C2/c	37.16	22.815	10.7854	90	93.092	90	[9]
5C	C2/c	39.7702	22.4499	10.8784	90	90.483	90	[9]
6C	P1	12.8761	16.5123	18.537	102.277	104.442	97.331	[9]
7C	P62c	16.4279	16.4279	21.9721	90	90	120	[9]
9C	$P2_1/n$	10.6473	19.677	48.7602	90	94.934	90	[9]
10C OTf	$P2_1/c$	18.8649	28.885	23.944	90	105.667	90	[9]
10C	C2/c	22.921	16.858	31.905	90	102.424	90	[9]
**ZrR-1**	Pm-3	26.8894	26.8894	26.8894	90	90	90	this work
**ZrR-2**	P-1	15.1314	15.5195	25.2801	98.108	95.711	112.659	this work

Table S3. The value of proton conductivity under different RH about **ZrR-1**.

**Humidity (%)**	**Conductivity (S cm^{-1})**
50	2.58×10^{-7}
60	4.24×10^{-7}
70	8.65×10^{-7}
80	5.14×10^{-6}
90	1.15×10^{-3}
98	6.80×10^{-3}

Table S4. The value of proton conductivity under different temperature about **ZrR-1**.

**Temperature (K)**	**Conductivity (S cm^{-1})**
303	6.80×10^{-3}
313	9.07×10^{-3}

323	1.05×10^{-2}
333	1.21×10^{-2}
343	1.22×10^{-2}
353	1.31×10^{-2}

Table S5. Proton conductivity about reported cage.

Compounds	Conductivity (S cm^{-1})	Conditions	Ea (eV)	ref
CPM-103a (single crystal)	5.8×10^{-2}	295.65 K, 98 %	0.66	[10]
Pd_6-1	0.78×10^{-3}	300 K, 46 %	0.36	[11]
Pd_6	0.22×10^{-3}	300 K, 46 %	0.245	[11]
In1-H	2.84×10^{-4}	298 K, 98%	0.28	[12]
Ga1-H	2.26×10^{-4}	298 K, 98%	0.34	[12]
MOP-1	1.41×10^{-3}	303.5 K, 98 %	0.225	[3]
Pd-2b	1.6×10^{-5}	273 K, 98 %	0.20	[13]
Pd-2c	1.1×10^{-3}	273 K, 98 %	0.39	[13]
$[Mn_6(CoL)_8](ClO_4)_{12}$	3.31×10^{-3}	343.5 K, 98 %	1.00	[14]
$[Mn_6(FeL)_8](ClO_4)_{12}$	1.05×10^{-4}	343.5 K, 98 %	0.78	[14]

Table S6. Proton conductivity about some other representative MOFs.

**Compounds**	**Medium**	**Conditions**	**Conductivity (S cm^{-1})**	E_a (eV)	**Ref**
Cu-TCPP	H_2O	25 °C, 98% RH	3.90×10^{-3}	0.28	[15]
KAUST-7'	H_2O	25 °C, 95% RH	6.70×10^{-3}	0.19	[16]
Mg-OBA	H_2O	25 °C, 95% RH	2.20×10^{-3}	0.13	[17]
UiO-66(Zr)-Br $Zr_6O_4(OH)_4$(BDC-Br)	H_2O	30 °C, 97% RH	2.23×10^{-7}	0.78	[18]
UiO-66(Zr)-Br $Zr_6O_4(OH)_4$(BDC-(NH_2))	H_2O	30 °C, 97% RH	1.40×10^{-5}	0.40	[18]
UiO-66(Zr)-Br $Zr_6O_4(OH)_4$(BDC)	H_2O	30 °C, 97% RH	7.54×10^{-6}	0.44	[18]
$[\{In_2(l\text{-}OH)_2(SO_4)_4\}\ \{(LH)_4\}\ nH_2O]_n$	H_2O	30 °C, 95% RH	1.10×10^{-5}	0.316	[19]
$[Zn(l\text{-}L_{Cl})\ (Cl)]\ (H_2O)_2$	H_2O	31 °C, 98% RH	4.45×10^{-5}	0.34	[20]
$[Zn(d\text{-}L_{Cl})\ (Cl)]\ (H_2O)_2$	H_2O	31 °C, 98% RH	4.42×10^{-5}	0.36	[20]
$Zn_3(L)\ (H_2O)_2\ 2H_2O$	H_2O	25 °C, 98% RH	3.50×10^{-5}	\	[21]
$\{[Er_3(PMPC)(OX)_3(H_2O)_7]\cdot 2H_2O\}n$	H_2O	25 °C, 97% RH	8.10×10^{-5}	0.33	[22]
$\{[K_8(PTC)_2(H_2O)_{1.5}]\cdot 4H_2O\}n$	H_2O	25 °C, 98% RH	1.00×10^{-3}	0.23	[23]
Li-HPAA $[Li_3(OOCCH(OH)PO_3)\ (H_2O)_4]\cdot H_2O$	H_2O, $-PO_3H$, $-COOH$	24 °C, 98% RH	1.10×10^{-4}	0.84	[24]
Na-HPAA $Na_2(OOCCH(OH)PO_3H)\ (H_2O)_4$	H_2O, $-PO_3H$, $-COOH$	24 °C, 98% RH	5.60×10^{-3}	0.39	[24]
K-HPAA $K_2\ (OOCCH(OH)PO_3H)\ (H_2O)_2$	H_2O, $-PO_3H$, $-COOH$	24 °C, 98% RH	1.30×10^{-3}	0.98	[24]
Cs-HPAA Cs $(HOOCCH(OH)\text{-}PO_3H)$	H_2O, $-PO_3H$, $-COOH$	24 °C, 98% RH	3.50×10^{-5}	0.40	[24]
CoLa-II $[CoLa(notpH)\ (H_2O)6]\ ClO_4\cdot 5H_2O$	H_2O, $-PO_3H$, ClO_4^-	25 °C, 95% RH	3.50×10^{-6}	0.34	[25]
CoLa-III $[H_3O]\ [CoLa(notp)\ (H_2O)_4]\ ClO_4\cdot 3H_2O$	H_2O, $-PO_3H$, ClO_4^-	25 °C, 95% RH	4.24×10^{-5}	0.28	[25]
MFM-500(Ni) $Ni_3(H_3L)_2(H_2O)_9\cdot(DMSO)_3$	H_2O, $-PO_3H$	25 °C, 98% RH	4.50×10^{-4}	0.43	[26]
MFM-500(Co) $Co_3(H_3L)_2(H_2O)_9\cdot(DMSO)_3$	H_2O, $-PO_3H$	25 °C, 98% RH	4.40×10^{-5}	\	[26]
$Rb_2(adp)[Zn_2(ox)_3]\cdot 3H_2O$	H_2O, $-COOH$	25 °C, 98% RH	4.30×10^{-5}	0.69	[27]
$K_2(adp)[Zn_2(ox)_3]\cdot 3H_2O$	H_2O, $-COOH$	25 °C, 98% RH	1.20×10^{-4}	0.63	[28]
$(NH_4)_2(adp)[Zn_2(ox)_3]\cdot 3H_2O$	H_2O, $-COOH$	25 °C, 98% RH	8.00×10^{-2}	0.63	[29]
$[Ba_2(L^1)\ (H_2O)_{1.5}(CO_2)\ (DMF)_{1.5}]$	H_2O, $-COOH$	25 °C, 99% RH	2.10×10^{-5}	0.63	[30]
$[Ba(H_2L^2)\ (H_2O)\ (DMF)]$	H_2O, $-COOH$	25 °C, 99% RH	5.10×10^{-5}	0.40	[30]
$[Ba_2(HL^3)\ (H_2O)_4]$	H_2O, $-COOH$	25 °C, 99% RH	2.90×10^{-5}	0.32	[30]
In-IA-2D-1	H_2O, $[(CH_3)_2NH_2]^+$	27 °C, 98% RH	3.40×10^{-3}	\	[31]
In-IA-2D-2	H_2O, DMF, $[(CH_3)_2NH_2]^+$	25 °C, 98% RH	2.60×10^{-5}	\	[31]
$[(CH_3)_2NH_2]$ [In(m-TTFTB)]	H_2O, $[(CH_3)_2NH_2]^+$	30 °C, 98% RH	6.60×10^{-4}	0.59	[32]
$[(CH_3)_2NH_2]$ [In (TTFOC)]	H_2O, $[(CH_3)_2NH_2]^+$	30 °C, 98% RH	1.30×10^{-2}	0.09	[32]
$(Me_2NH_2)\ (Me_2NH)\ [In(mdhbqdc)_2]$	H_2O, $[(CH_3)_2NH_2]^+$	30 °C, 95% RH	2.10×10^{-4}	0.73	[33]
ZrPP-1	H_2O, $[(CH_3)_2NH_2]^+$	25 °C, 98% RH	8.00×10^{-3}	0.21	[34]

ZrPP-2	H_2O, $[(CH_3)_2NH_2]^+$	25 °C, 98% RH	4.2×10^{-3}	0.23	[34]
Mg_2(DOBDC)(Urea)$_2$	Urea	25 °C, 95% RH	2.64×10^{-2}	\	[35]
Ni_2(DOBDC)(Urea)$_2$	Urea	25 °C, 95% RH	6.19×10^{-4}	\	[35]

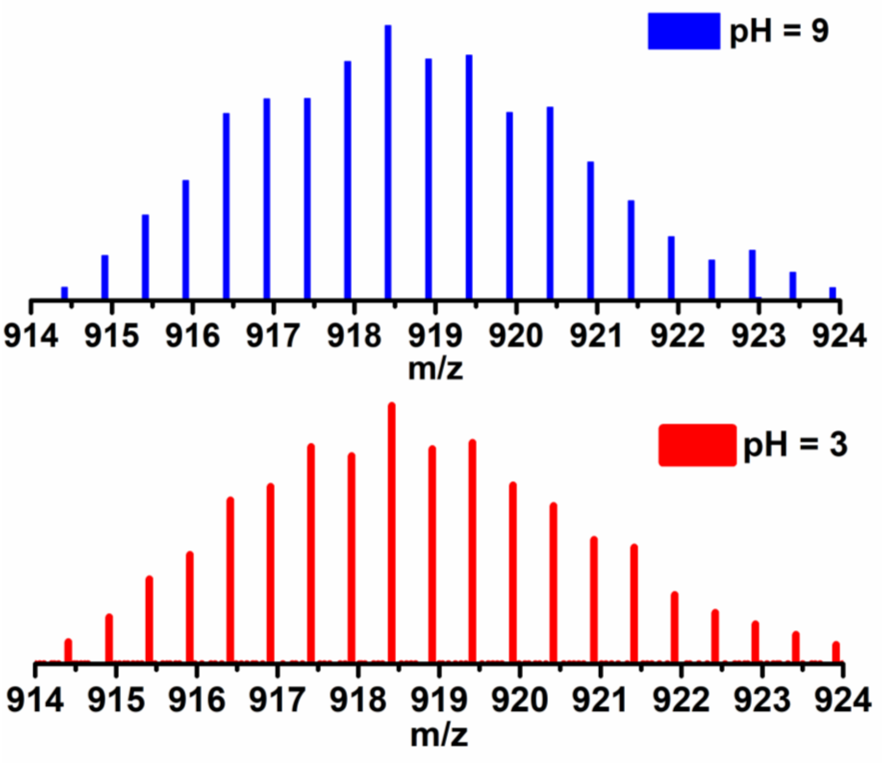

Figure S1. ESI-TOF-MS spectra under different pH values.

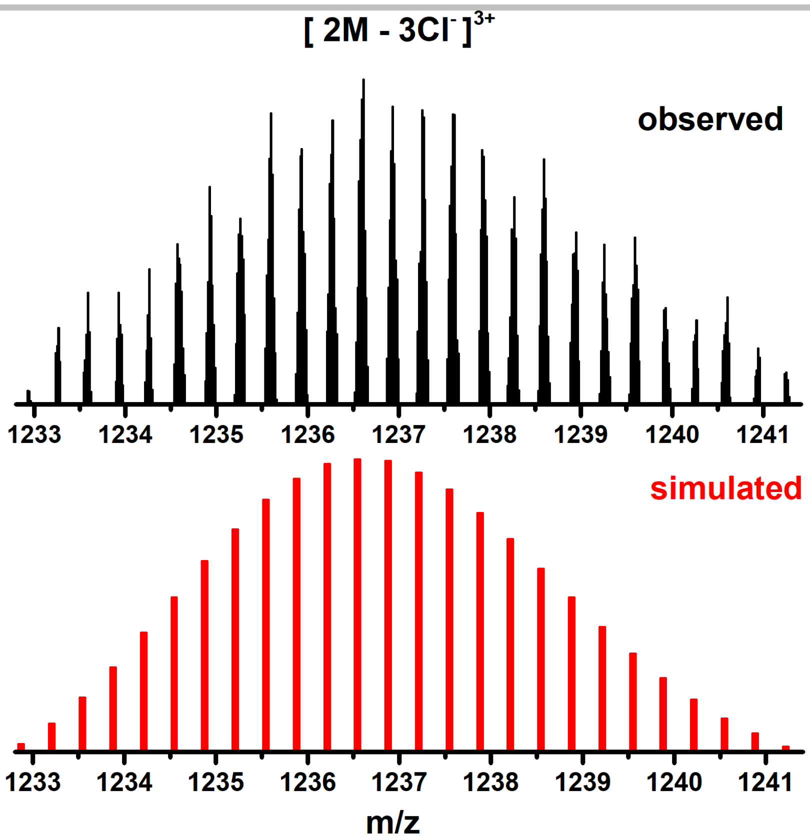

Figure S2. ESI-TOF-MS spectra of supramolecular dimer.

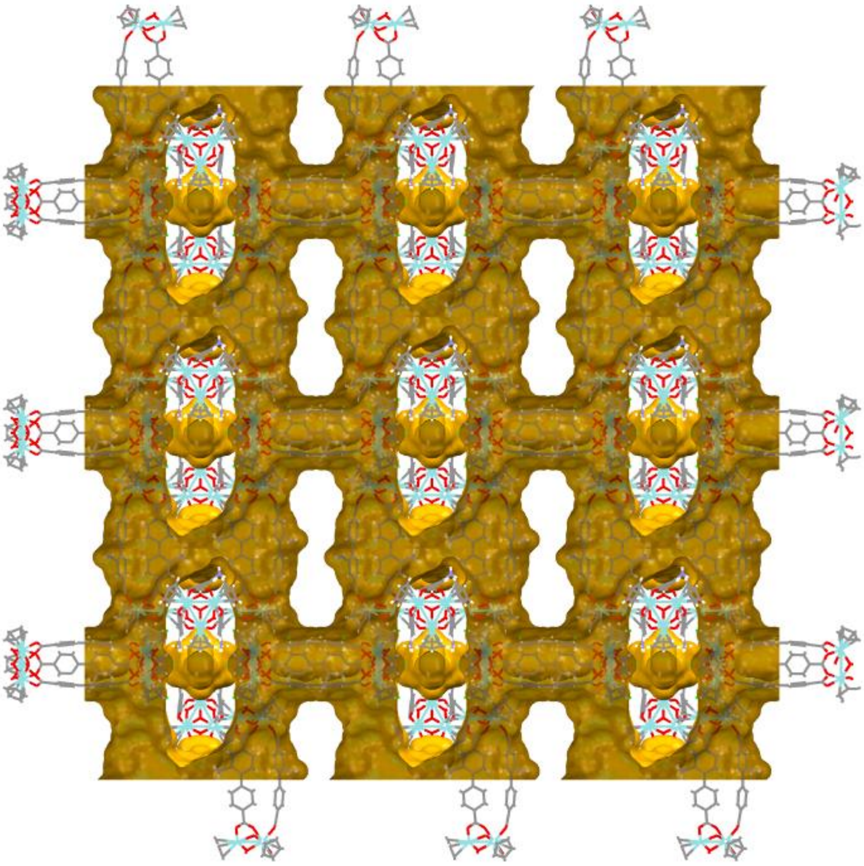

Figure S3. Channels structure of **ZrR-1**.

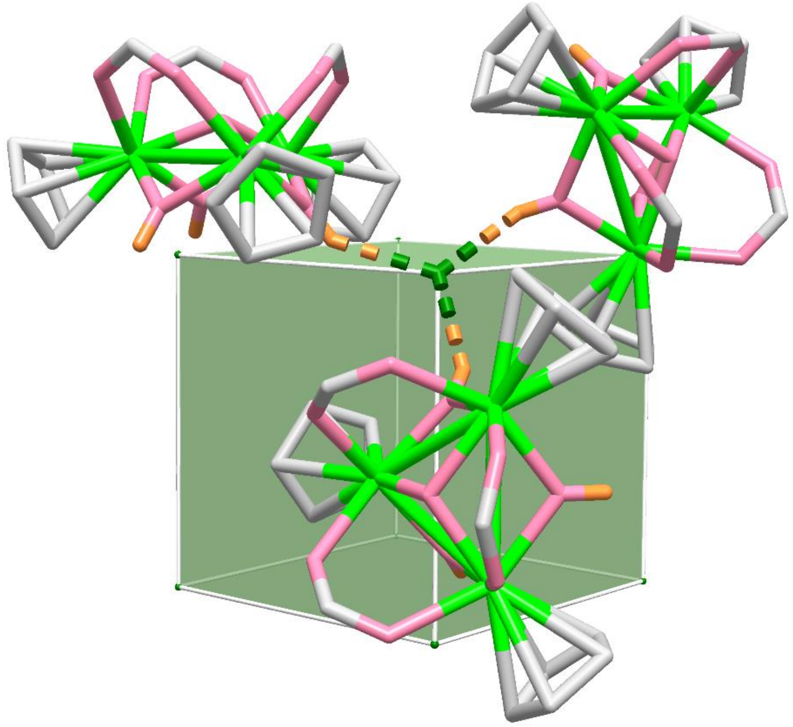

Figure S4. The Cl^- at the vertex forms hydrogen bonds with three OH groups.

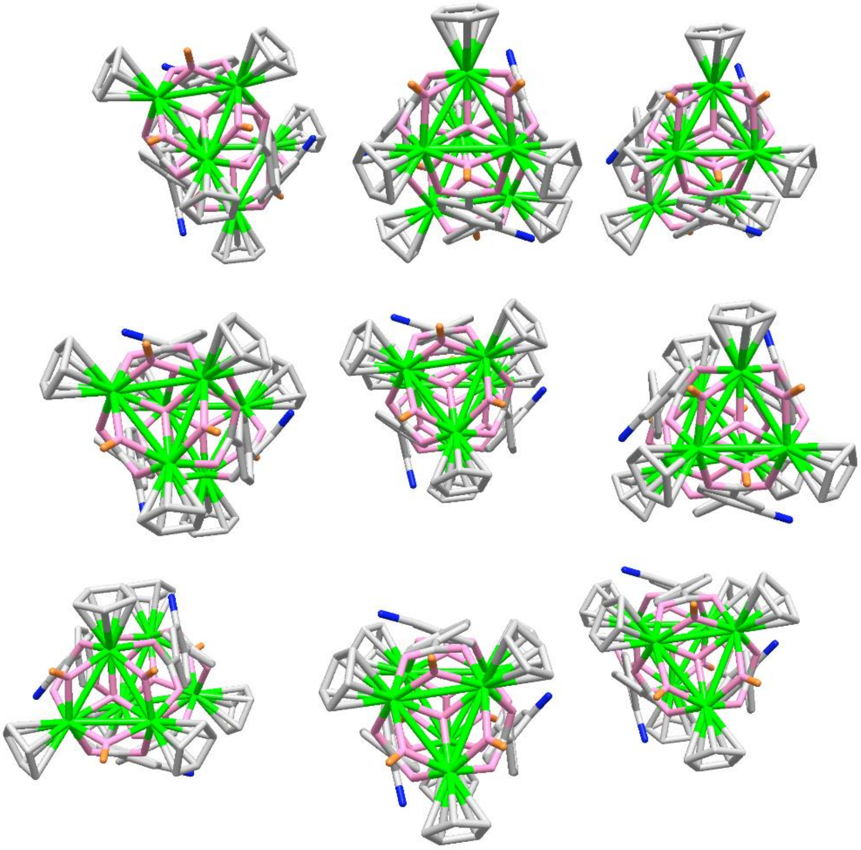

Figure S5. Parallel Arrangement of Zr-Cages in **ZrR-2**.

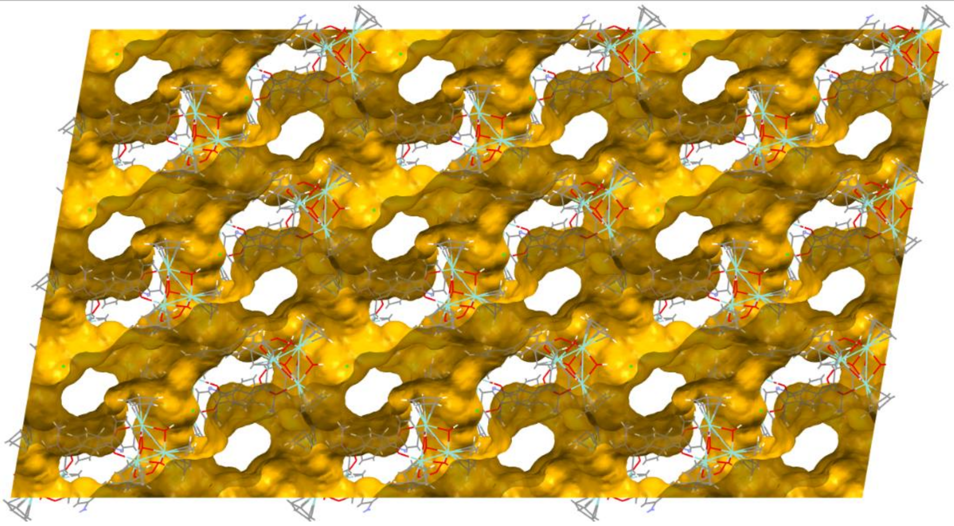

Figure S6. Channels structure of **ZrR-2**.

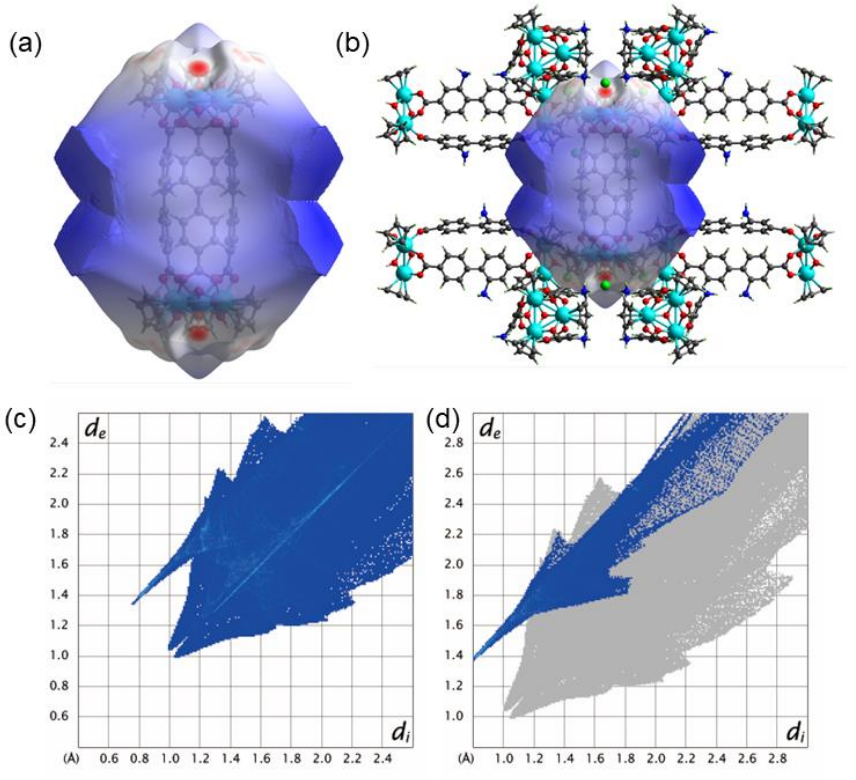

Figure S7. (a) The Hirshfeld surface of **ZrR-1**; (b) Each V_2L_3 **ZrR-1** surrounded by another 12 Zr-Cages; (c) Fingerprint plot of **ZrR-1**; (d) Decomposed (O−H···Cl⁻) fingerprint plot of **ZrR-1**.

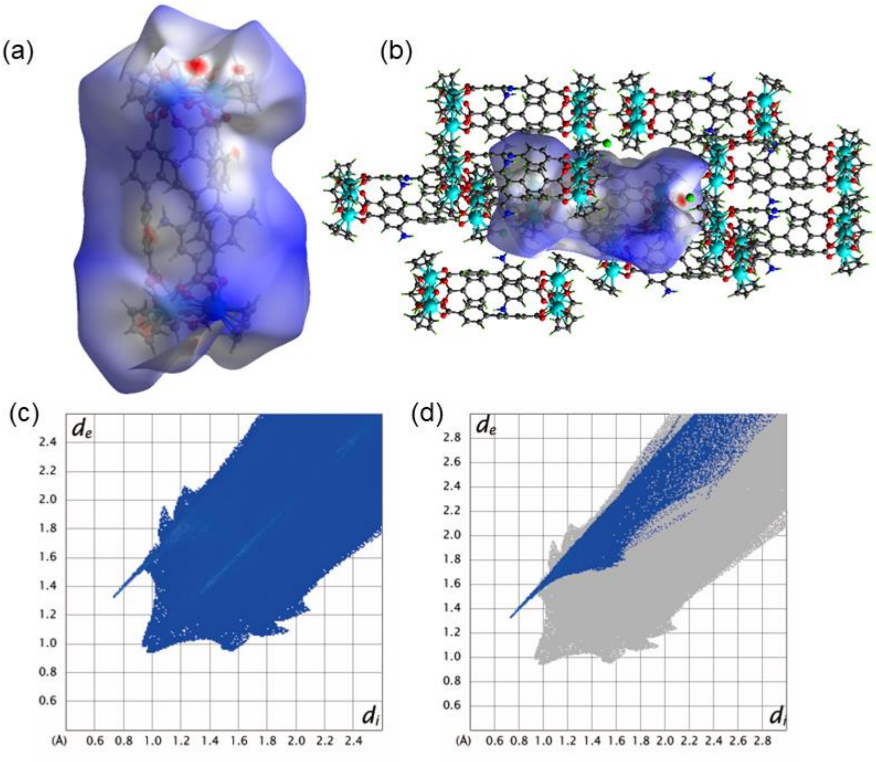

Figure S8. (a) The Hirshfeld surface of **ZrR-2**; (b) Each V_2L_3 **ZrR-2** surrounded by another 8 Zr-Cages; (c) Fingerprint plot of **ZrR-2**; (d) Decomposed (O−H···Cl⁻) fingerprint plot of **ZrR-2**.

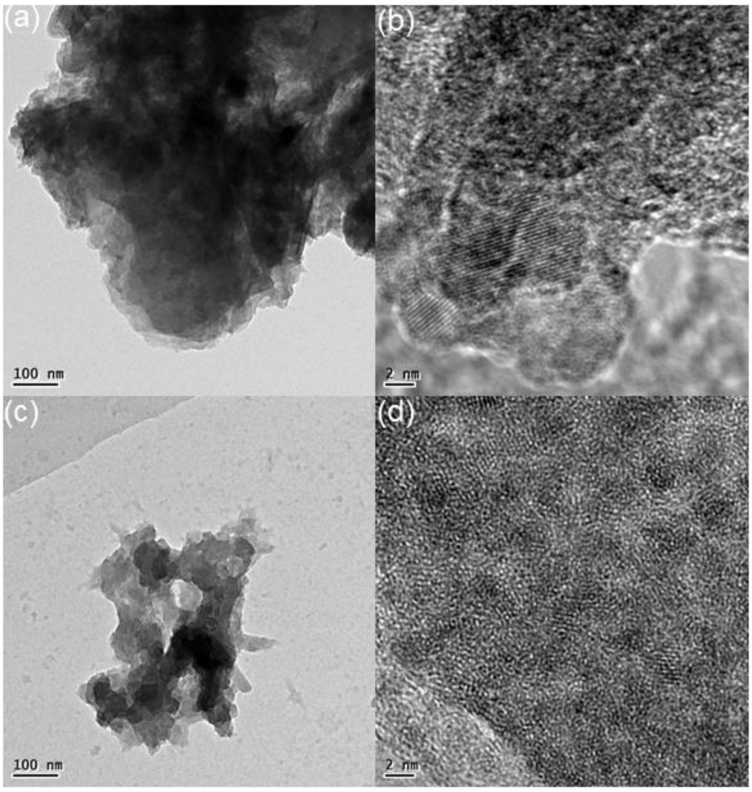

Figure S9. TEM images of **ZrR-1** (a and b) and **ZrR-2** (c and d).

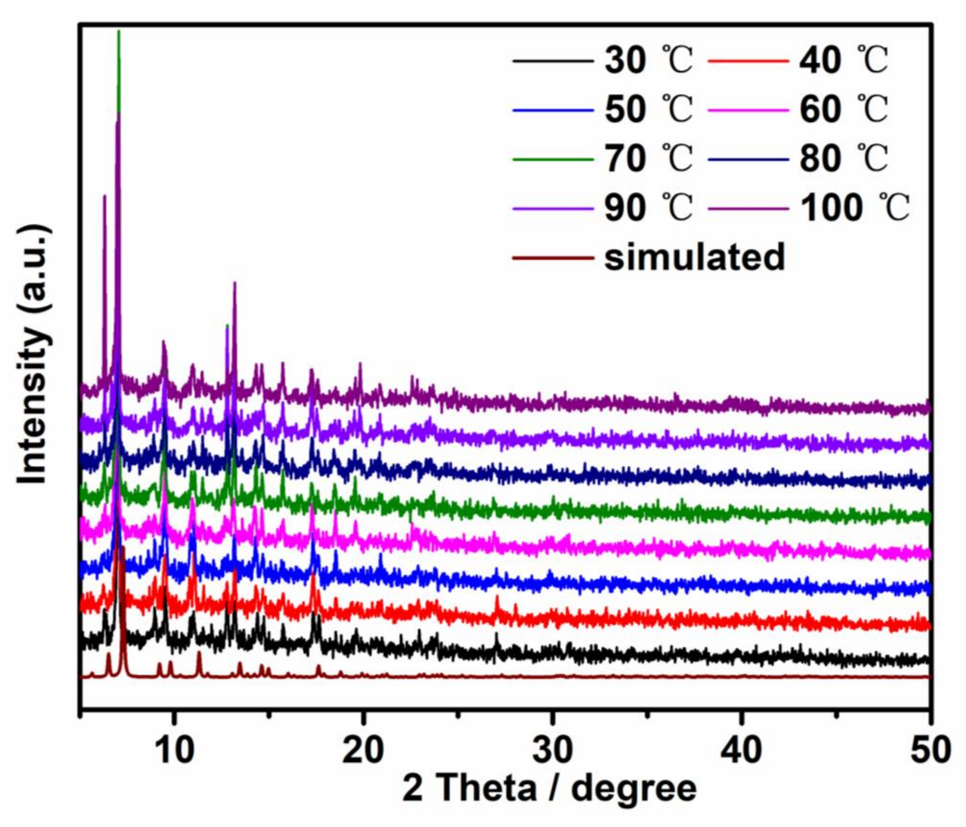

Figure S10. PXRD patterns of **ZrR-1** in different temperatures.

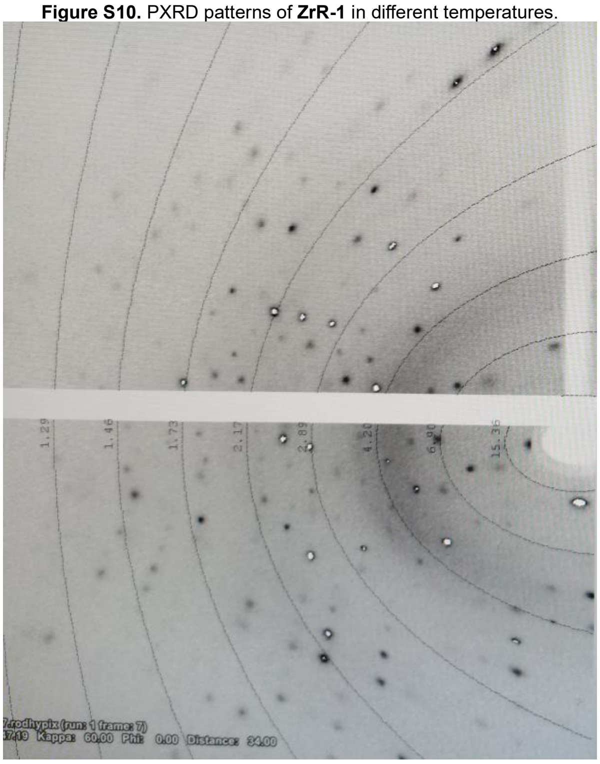

Figure S11. Diffraction spots of **ZrR-1** after exposure to a humid environment for 6 months.

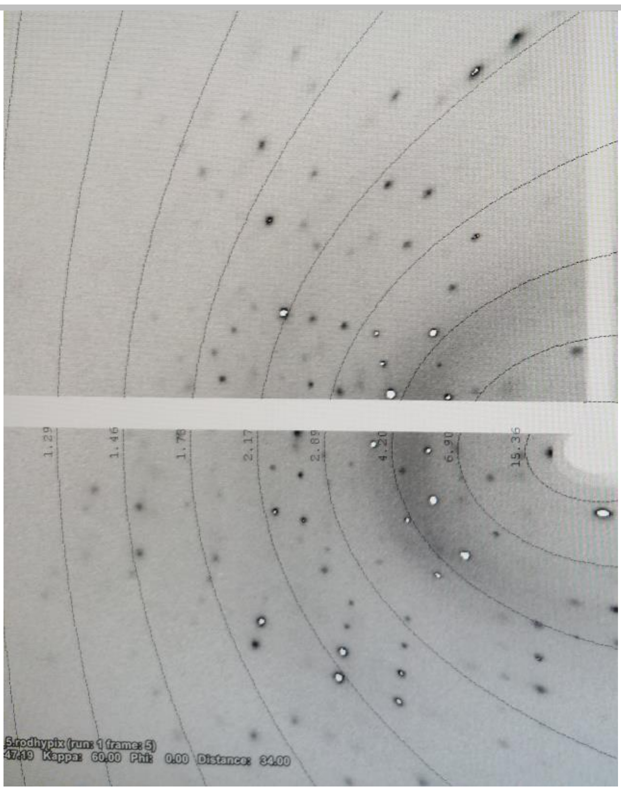

Figure S12. Diffraction spots of **ZrR-1** after gas adsorption experiment.

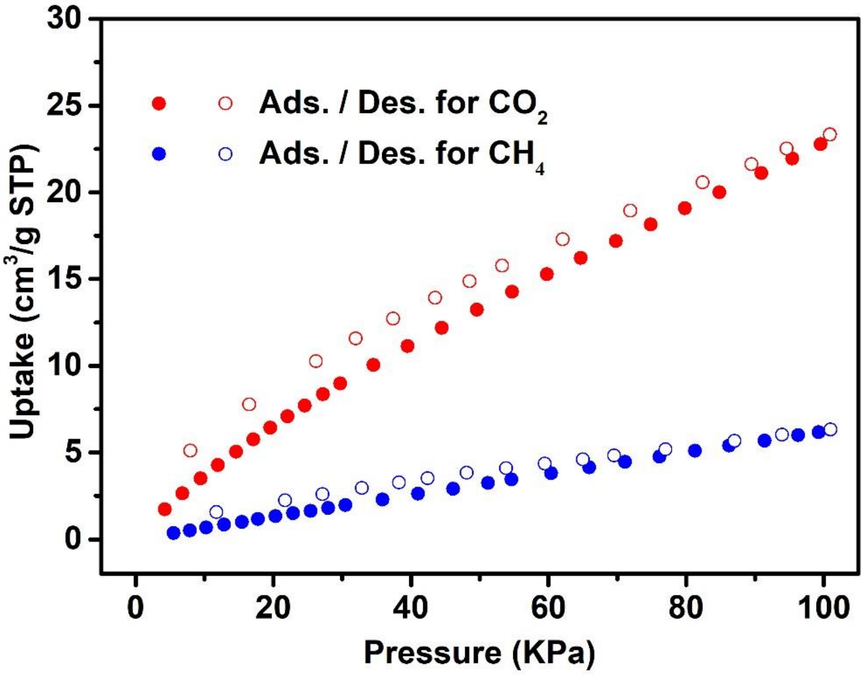

Figure S13. CO_2 and CH_4 sorption isotherm for **ZrR-1**.

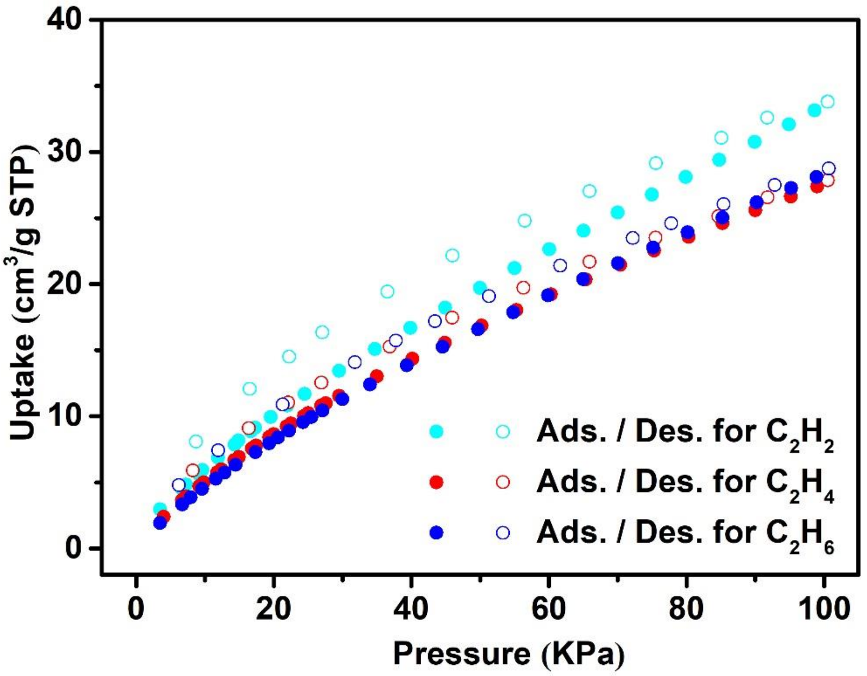

Figure S14. C_2H_4, C_2H_4 and C_2H_6 sorption isotherm for **ZrR-1**.

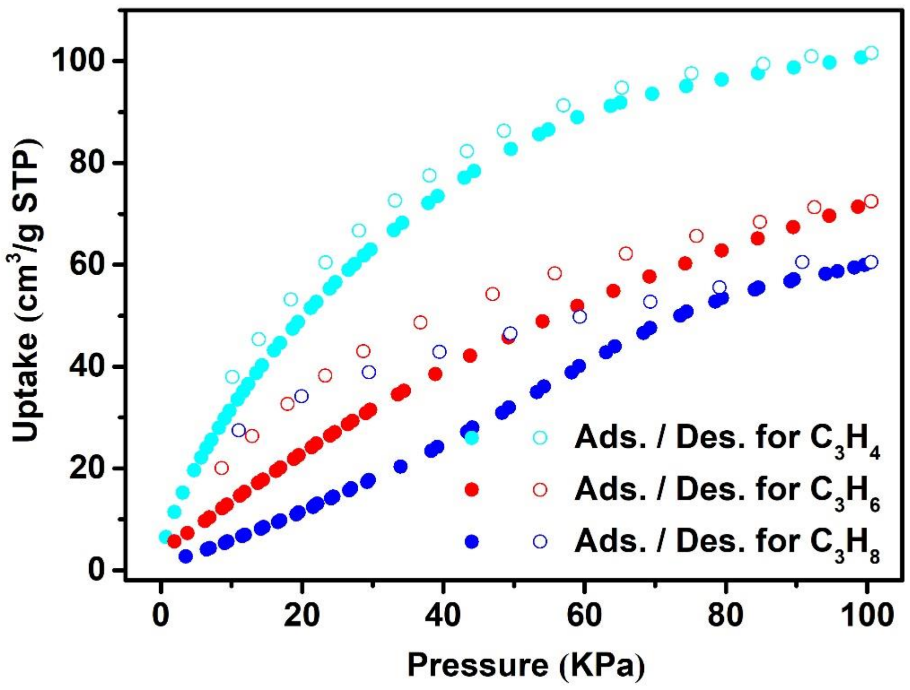

Figure S15. C_3H_4, C_3H_6 and C_3H_8 sorption isotherm for **ZrR-1**.

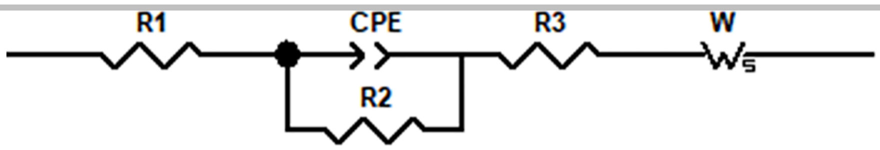

Element	Value
R1	50
CPE-T	8.5×10^{-8}
CPE-P	0.758
R2	3930
R3	1011
W-R	34603
W-T	2.102
W-P	0.355

Figure S16. Parameters derived from the equivalent circuit fitting of Nyquist plots in Figure 5b.

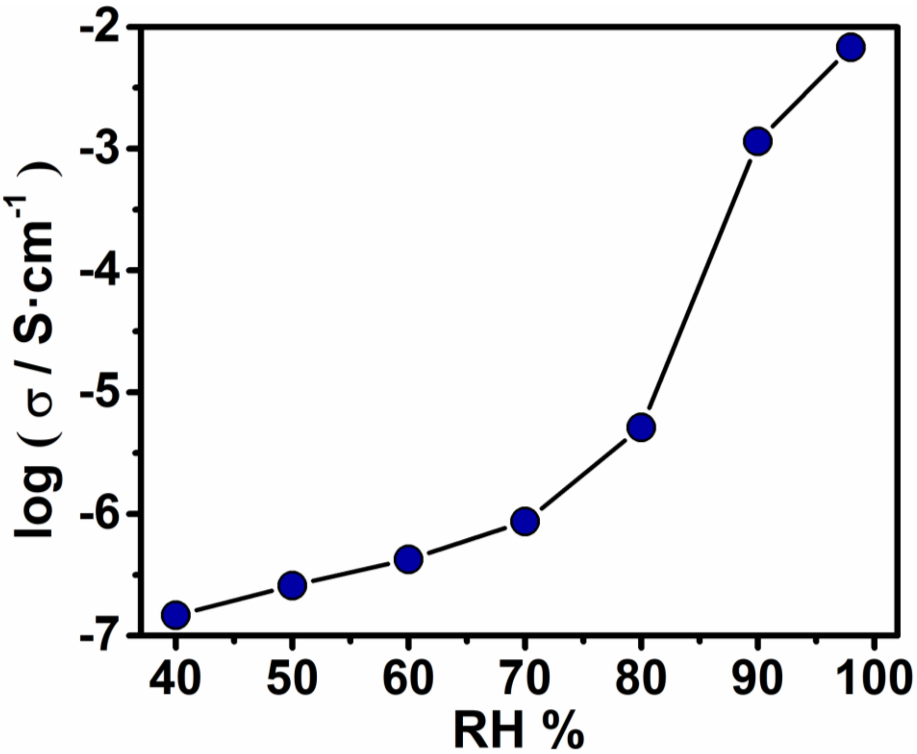

Figure S17. Humidity-dependent proton conductivity of **ZrR-1** at 303 K.

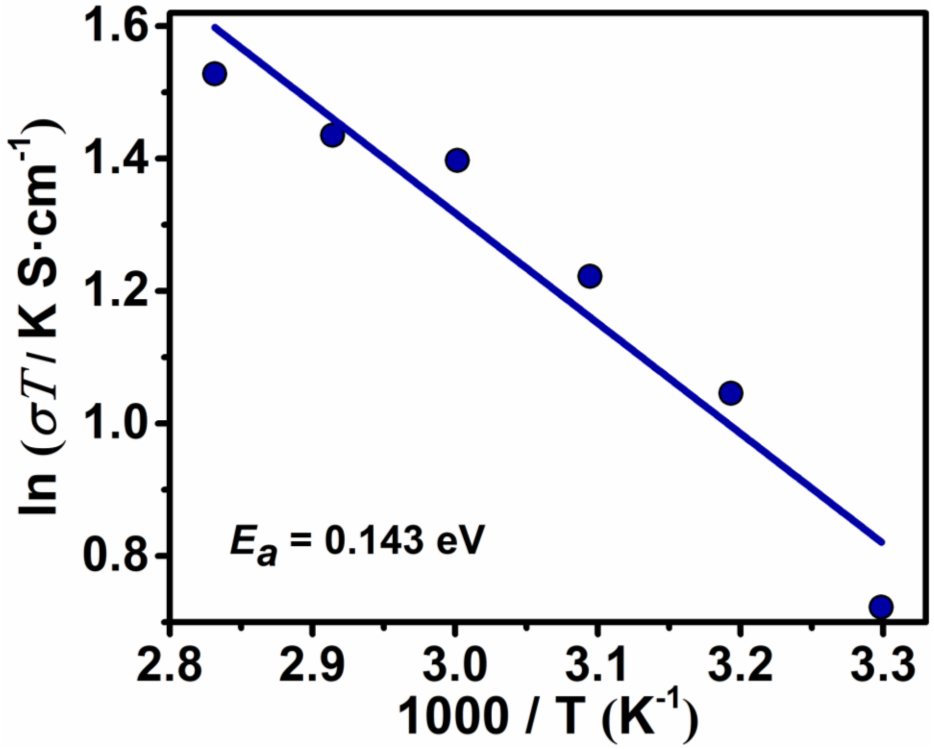

Figure S18. Arrhenius plots of conductivities of **ZrR-1** from 303 to 353 K at 98% RH.

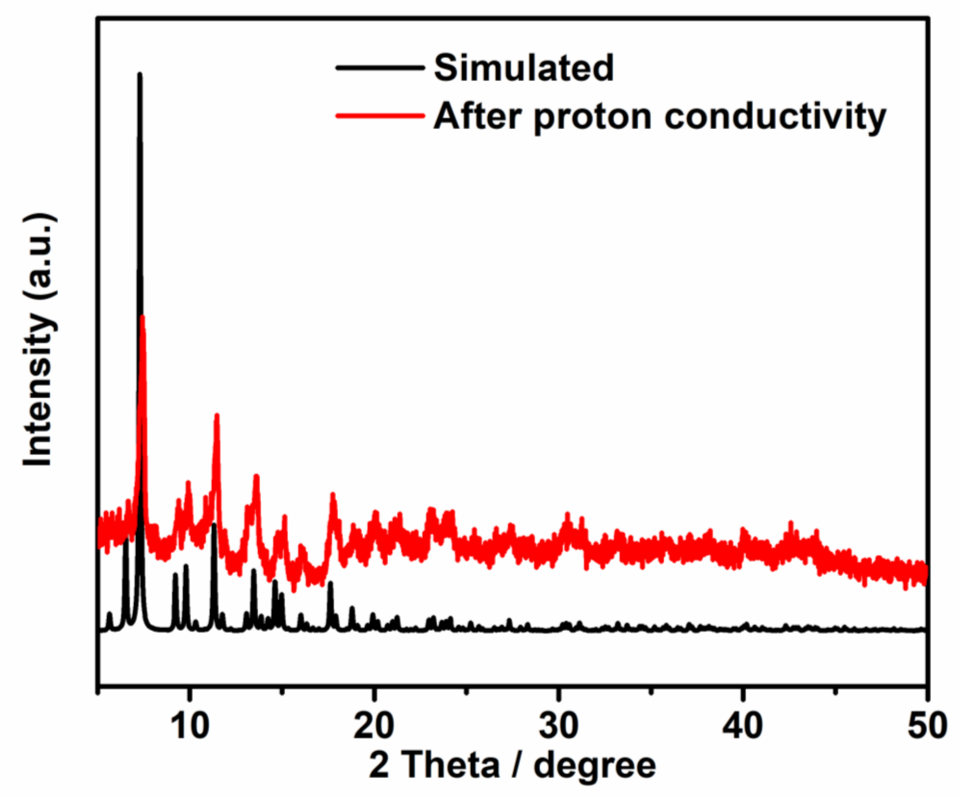

Figure S19. PXRD patterns of **ZrR-1** after proton conductivity.

References

[1] S. Chen, S. Cheng, L. Zhao, C. Sun, C. Qin, Z. Su, *New J. Chem.* **2020**, *44*, 21255-21260.
[2] X. Chen, S. B. Li, Z. Y. Liu, Y. T. Zhang, *J. Solid State Chem.* **2021**, *296*, 121998-122002.
[3] W.-H. Xing, H.-Y. Li, X.-Y. Dong, S.-Q. Zang, *J. Mater. Chem. A.* **2018**, *6*, 7724-7730.
[4] Y. H. Zou, Q. J. Wu, Q. Yin, Y. B. Huang, R. Cao, *Inorg. Chem.* **2021**, *60*, 2112-2116.
[5] S. Lee, J. H. Lee, J. C. Kim, S. Lee, S. K. Kwak, W. Choe, *ACS Appl. Mater. Interfaces* **2018**, *10*, 8685-8691.
[6] Y. Jiang, P. Tan, S.-C. Qi, C. Gu, S.-S. Peng, F. Wu, X.-Q. Liu, L.-B. Sun, *CCS Chemistry* **2021**, *3*, 1659-1668.
[7] W. J. Shi, D. Liu, X. Li, S. Bai, Y. Y. Wang, Y. F. Han, *Chem. Eur. J.* **2021**, *27*, 7853-7861.
[8] H. Yang, M. Li, Y. Cai, M. Zhou, G. Liu, *Cryst. Growth Des.* **2021**, *21*, 6642-6647.
[9] E. J. Gosselin, G. E. Decker, B. W. McNichols, J. E. Baumann, G. P. A. Yap, A. Sellinger, E. D. Bloch, *Chem. Mater.* **2020**, *32*, 5872-5878.
[10] Q. G. Zhai, C. Mao, X. Zhao, Q. Lin, F. Bu, X. Chen, X. Bu, P. Feng, *Angew. Chem. Int. Ed.* **2015**, *54*, 7886-7890.
[11] D. Samanta, P. S. Mukherjee, *Chem. Commun.* **2014**, *50*, 1595-1598.
[12] X. Du, R. Fan, L. Qiang, Y. Song, K. Xing, W. Chen, P. Wang, Y. Yang, *Inorg. Chem.* **2017**, *56*, 3429-3439.
[13] D. Samanta, P. S. Mukherjee, *Chem. Eur. J.* **2014**, *20*, 12483-12492.
[14] R. Saha, D. Samanta, A. J. Bhattacharyya, P. S. Mukherjee, *Chem. Eur. J.* **2017**, *23*, 8980-8986.
[15] G. Xu, K. Otsubo, T. Yamada, S. Sakaida, H. Kitagawa, *J. Am. Chem. Soc.* **2013**, *135*, 7438-7441.
[16] P. G. M. Mileo, K. Adil, L. Davis, A. Cadiau, Y. Belmabkhout, H. Aggarwal, G. Maurin, M. Eddaoudi, S. Devautour-Vinot, *J. Am. Chem. Soc.* **2018**, *140*, 13156-13160.
[17] S. Chand, S. C. Pal, D.-W. Lim, K. Otsubo, A. Pal, H. Kitagawa, M. C. Das, *ACS Mater. Lett.* **2020**, *2*, 1343-1350.
[18] F. Yang, H. Huang, X. Wang, F. Li, Y. Gong, C. Zhong, J.-R. Li, *Cryst. Growth Des.* **2015**, *15*, 5827-5833.
[19] B. Manna, B. Anothumakkool, A. V. Desai, P. Samanta, S. Kurungot, S. K. Ghosh, *Inorg. Chem.* **2015**, *54*, 5366-5371.
[20] S. C. Sahoo, T. Kundu, R. Banerjee, *J. Am. Chem. Soc.* **2011**, *133*, 17950-17958.
[21] J. M. Taylor, R. K. Mah, I. L. Moudrakovski, C. I. Ratcliffe, R. Vaidhyanathan, G. K. H. Shimizu, *J. Am. Chem. Soc.* **2010**, *132*, 14055-14057.
[22] X. Liang, K. Cai, F. Zhang, J. Liu, G. Zhu, *J. Mater. Chem. A.* **2017**, *5*, 25350-25358.
[23] N. Sikdar, D. Dutta, R. Haldar, T. Ray, A. Hazra, A. J. Bhattacharyya, T. K. Maji, *J. Phys. Chem. C* **2016**, *120*, 13622-13629.
[24] M. Bazaga-García, M. Papadaki, R. M. P. Colodrero, P. Olivera-Pastor, E. R. Losilla, B. Nieto-Ortega, M. Á. G. Aranda, D. Choquesillo-Lazarte, A. Cabeza, K. D. Demadis, *Chem. Mater.* **2015**, *27*, 424-435.
[25] S.-S. Bao, K. Otsubo, J. M. Taylor, Z. Jiang, L.-M. Zheng, H. Kitagawa, *J. Am. Chem. Soc.* **2014**, *136*, 9292-9295.
[26] S. Pili, S. P. Argent, C. G. Morris, P. Rought, V. García-Sakai, I. P. Silverwood, T. L. Easun, M. Li, M. R. Warren, C. A. Murray, C. C. Tang, S. Yang, M. Schröder, *J. Am. Chem. Soc.* **2016**, *138*, 6352-6355.
[27] M. Sadakiyo, T. Yamada, H. Kitagawa, *Inorg. Chem. Commun.* **2016**, *72*, 138-140.
[28] M. Sadakiyo, T. Yamada, H. Kitagawa, *J. Am. Chem. Soc.* **2014**, *136*, 13166-13169.
[29] M. Sadakiyo, T. Yamada, H. Kitagawa, *J. Am. Chem. Soc.* **2009**, *131*, 9906-9907.
[30] P. Rought, C. Marsh, S. Pili, I. P. Silverwood, V. G. Sakai, M. Li, M. S. Brown, S. P. Argent, I. Vitorica-Yrezabal, G. Whitehead, M. R. Warren, S. Yang, M. Schröder, *Chem. Sci.* **2019**, *10*, 1492-1499.
[31] T. Panda, T. Kundu, R. Banerjee, *Chem. Commun.* **2013**, *49*, 6197-6199.
[32] J. Su, W. He, X.-M. Li, L. Sun, H.-Y. Wang, Y.-Q. Lan, M. Ding, J.-L. Zuo, *Matter* **2020**, *2*, 711-722.
[33] H. Gao, Y.-B. He, J.-J. Hou, Q.-G. Zhai, X.-M. Zhang, *ACS Appl. Mater. Interfaces* **2020**, *12*, 41605-41612.
[34] E.-X. Chen, G. Xu, Q. Lin, *Inorg. Chem.* **2019**, *58*, 3569-3573.
[35] M. K. Sarango-Ramírez, D.-W. Lim, D. I. Kolokolov, A. E. Khudozhitkov, A. G. Stepanov, H. Kitagawa, *J. Am. Chem. Soc.* **2020**, *142*, 6861-6865.
