# Supplementary material for: Hierarchical Self‐Assembly of Capsule‐Shaped Zirconium Coordination Cages with Quaternary Structure
Source: Adv Sci (Weinh). 2024 Jan 16;11(11):2308445. doi: 10.1002/advs.202308445 (PMC10953209; doi:10.1002/advs.202308445)

checkCIF/PLATON report

You have not supplied any structure factors. As a result the full set of tests cannot be run.

THIS REPORT IS FOR GUIDANCE ONLY. IF USED AS PART OF A REVIEW PROCEDURE FOR PUBLICATION, IT SHOULD NOT REPLACE THE EXPERTISE OF AN EXPERIENCED CRYSTALLOGRAPHIC REFEREE.

No syntax errors found.   CIF dictionary   Interpreting this report

Datablock: ZrR-1

```
Bond precision:   C-C = 0.0098 A          Wavelength=1.54184

Cell:              a=26.8894(2)      b=26.8894(2)      c=26.8894(2)
                   alpha=90          beta=90           gamma=90
Temperature:       100 K
```

	Calculated	Reported
Volume	19442.1(4)	19442.1(4)
Space group	P m -3	P m -3
Hall group	-P 2 2 3	-P 2 2 3
Moiety formula	C72 H62 N3 O20 Zr6, 2(Cl) [+ solvent]	?
Sum formula	C72 H62 Cl2 N3 O20 Zr6 [+ solvent]	C72 H62 Cl2 N3 O20 Zr6
Mr	1907.47	1907.46
Dx,g cm-3	0.978	0.977
Z	6	6
Mu (mm-1)	4.549	4.549
F000	5694.0	5694.0
F000’	5705.11	
h,k,lmax	33,33,33	30,30,33
Nref	7286	6950
Tmin,Tmax	0.608,0.635	0.879,1.000
Tmin’	0.552	

```
Correction method= # Reported T Limits: Tmin=0.879 Tmax=1.000
AbsCorr = MULTI-SCAN

Data completeness= 0.954            Theta(max)= 76.890
```

R(reflections)= 0.0596(5294)

wR2(reflections)= 0.1994(6950)

S = 1.034 Npar= 313

The following ALERTS were generated. Each ALERT has the format
test-name_ALERT_alert-type_alert-level.
Click on the hyperlinks for more details of the test.

Alert level B

```
PLAT094_ALERT_2_B Ratio of Maximum / Minimum Residual Density ....       4.33 Report
```

Alert level C

```
PLAT213_ALERT_2_C Atom N1              has ADP max/min Ratio .....        3.2 prolat
PLAT222_ALERT_3_C NonSolvent Resd 1   H   Uiso(max)/Uiso(min) Range       4.1 Ratio
PLAT241_ALERT_2_C High   'MainMol' Ueq as Compared to Neighbors of         C8 Check
PLAT242_ALERT_2_C Low    'MainMol' Ueq as Compared to Neighbors of        C13 Check
PLAT342_ALERT_3_C Low Bond Precision on  C-C Bonds ...............    0.00975 Ang.
PLAT420_ALERT_2_C D-H Bond Without Acceptor  N2       --H2B      .     Please Check
```

Alert level G

```
PLAT002_ALERT_2_G Number of Distance or Angle Restraints on AtSite          2 Note
PLAT003_ALERT_2_G Number of Uiso or Uij Restrained non-H Atoms ...          1 Report
PLAT007_ALERT_5_G Number of Unrefined Donor-H Atoms .............           6 Report
PLAT012_ALERT_1_G N.O.K.   _shelx_res_checksum Found in CIF ......     Please Check
PLAT083_ALERT_2_G SHELXL Second Parameter in WGHT  Unusually Large      38.33 Why ?
PLAT172_ALERT_4_G The CIF-Embedded .res File Contains DFIX Records          1 Report
PLAT178_ALERT_4_G The CIF-Embedded .res File Contains SIMU Records          1 Report
PLAT186_ALERT_4_G The CIF-Embedded .res File Contains ISOR Records          1 Report
PLAT187_ALERT_4_G The CIF-Embedded .res File Contains RIGU Records          1 Report
PLAT300_ALERT_4_G Atom Site Occupancy of N2        Constrained at         0.5 Check
PLAT300_ALERT_4_G Atom Site Occupancy of C11       Constrained at         0.5 Check
PLAT300_ALERT_4_G Atom Site Occupancy of C12       Constrained at         0.5 Check
PLAT300_ALERT_4_G Atom Site Occupancy of C14       Constrained at         0.5 Check
PLAT300_ALERT_4_G Atom Site Occupancy of C15       Constrained at         0.5 Check
PLAT300_ALERT_4_G Atom Site Occupancy of C19       Constrained at         0.5 Check
PLAT300_ALERT_4_G Atom Site Occupancy of C20       Constrained at         0.5 Check
PLAT300_ALERT_4_G Atom Site Occupancy of C21       Constrained at         0.5 Check
PLAT300_ALERT_4_G Atom Site Occupancy of C22       Constrained at         0.5 Check
PLAT300_ALERT_4_G Atom Site Occupancy of C23       Constrained at         0.5 Check
PLAT300_ALERT_4_G Atom Site Occupancy of C24       Constrained at         0.5 Check
PLAT300_ALERT_4_G Atom Site Occupancy of C25       Constrained at         0.5 Check
PLAT300_ALERT_4_G Atom Site Occupancy of C26       Constrained at         0.5 Check
PLAT300_ALERT_4_G Atom Site Occupancy of N1        Constrained at        0.25 Check
PLAT300_ALERT_4_G Atom Site Occupancy of H2A       Constrained at         0.5 Check
PLAT300_ALERT_4_G Atom Site Occupancy of H2B       Constrained at         0.5 Check
PLAT300_ALERT_4_G Atom Site Occupancy of H11       Constrained at         0.5 Check
PLAT300_ALERT_4_G Atom Site Occupancy of H12       Constrained at         0.5 Check
PLAT300_ALERT_4_G Atom Site Occupancy of H15       Constrained at         0.5 Check
PLAT300_ALERT_4_G Atom Site Occupancy of H19       Constrained at         0.5 Check
PLAT300_ALERT_4_G Atom Site Occupancy of H20       Constrained at         0.5 Check
PLAT300_ALERT_4_G Atom Site Occupancy of H22       Constrained at         0.5 Check
PLAT300_ALERT_4_G Atom Site Occupancy of H23       Constrained at         0.5 Check
```

```
PLAT300_ALERT_4_G Atom Site Occupancy of H24       Constrained at        0.5 Check
PLAT300_ALERT_4_G Atom Site Occupancy of H25       Constrained at        0.5 Check
PLAT300_ALERT_4_G Atom Site Occupancy of H26       Constrained at        0.5 Check
PLAT300_ALERT_4_G Atom Site Occupancy of H1A       Constrained at       0.25 Check
PLAT300_ALERT_4_G Atom Site Occupancy of H1B       Constrained at       0.25 Check
PLAT300_ALERT_4_G Atom Site Occupancy of Cl1       Constrained at       0.75 Check
PLAT300_ALERT_4_G Atom Site Occupancy of Cl2       Constrained at        0.5 Check
PLAT301_ALERT_3_G Main Residue  Disorder .............(Resd  1  )        27% Note
PLAT302_ALERT_4_G Anion/Solvent/Minor-Residue Disorder (Resd  2  )       100% Note
PLAT302_ALERT_4_G Anion/Solvent/Minor-Residue Disorder (Resd  3  )       100% Note
PLAT304_ALERT_4_G Non-Integer Number of Atoms in ..... (Resd  2  )       0.25 Check
PLAT304_ALERT_4_G Non-Integer Number of Atoms in ..... (Resd  3  )       0.25 Check
PLAT432_ALERT_2_G Short Inter X...Y Contact  C2       ..C16      .       3.18 Ang.
                                                     y,z,x  =      3_555 Check
PLAT432_ALERT_2_G Short Inter X...Y Contact  C3       ..C16      .       3.18 Ang.
                                                     y,z,x  =      3_555 Check
PLAT606_ALERT_4_G Solvent Accessible VOID(S) in Structure ........          ! Info
PLAT764_ALERT_4_G Overcomplete CIF Bond List Detected (Rep/Expd) .       1.28 Ratio
PLAT779_ALERT_4_G Suspect or Irrelevant (Bond) Angle(s) in CIF ...      37.00 Deg.
              C15  -C10  -C11     1_555   1_555  22_556 ......     #  170 Check
PLAT779_ALERT_4_G Suspect or Irrelevant (Bond) Angle(s) in CIF ...      37.00 Deg.
              C15  -C10  -C11    22_556   1_555   1_555 ......     #  171 Check
PLAT779_ALERT_4_G Suspect or Irrelevant (Bond) Angle(s) in CIF ...      33.30 Deg.
              C12  -C11  -C14     1_555   1_555  22_556 ......     #  182 Check
PLAT779_ALERT_4_G Suspect or Irrelevant (Bond) Angle(s) in CIF ...      32.90 Deg.
              C11  -C12  -C15     1_555   1_555  22_556 ......     #  188 Check
PLAT779_ALERT_4_G Suspect or Irrelevant (Bond) Angle(s) in CIF ...      38.40 Deg.
              C14  -C13  -C12    22_556   1_555   1_555 ......     #  191 Check
PLAT779_ALERT_4_G Suspect or Irrelevant (Bond) Angle(s) in CIF ...      38.40 Deg.
              C14  -C13  -C12     1_555   1_555  22_556 ......     #  194 Check
PLAT779_ALERT_4_G Suspect or Irrelevant (Bond) Angle(s) in CIF ...      31.80 Deg.
              C15  -C14  -C11     1_555   1_555  22_556 ......     #  207 Check
PLAT779_ALERT_4_G Suspect or Irrelevant (Bond) Angle(s) in CIF ...      34.40 Deg.
              C14  -C15  -C12     1_555   1_555  22_556 ......     #  214 Check
PLAT794_ALERT_5_G Tentative Bond Valency for Zr1       (IV)      .       4.09 Info
PLAT794_ALERT_5_G Tentative Bond Valency for Zr2       (IV)      .       4.18 Info
PLAT811_ALERT_5_G No ADDSYM Analysis: Too Many Excluded Atoms ....          ! Info
PLAT860_ALERT_3_G Number of Least-Squares Restraints ............           7 Note
PLAT869_ALERT_4_G ALERTS Related to the Use of SQUEEZE  Suppressed          ! Info
PLAT883_ALERT_1_G No Info/Value for _atom_sites_solution_primary .     Please Do !
PLAT933_ALERT_2_G Number of HKL-OMIT Records in Embedded .res File          4 Note
PLAT941_ALERT_3_G Average HKL Measurement Multiplicity ...........        3.7 Low
PLAT950_ALERT_5_G Calculated (ThMax) and CIF-Reported Hmax Differ           3 Units
PLAT951_ALERT_5_G Calculated (ThMax) and CIF-Reported Kmax Differ           3 Units
PLAT965_ALERT_2_G The SHELXL WEIGHT Optimisation has not Converged     Please Check
```

---

0 **ALERT level A** = Most likely a serious problem - resolve or explain
1 **ALERT level B** = A potentially serious problem, consider carefully
6 **ALERT level C** = Check. Ensure it is not caused by an omission or oversight
67 **ALERT level G** = General information/check it is not something unexpected

2 ALERT type 1 CIF construction/syntax error, inconsistent or missing data
12 ALERT type 2 Indicator that the structure model may be wrong or deficient
5 ALERT type 3 Indicator that the structure quality may be low
49 ALERT type 4 Improvement, methodology, query or suggestion
6 ALERT type 5 Informative message, check

It is advisable to attempt to resolve as many as possible of the alerts in all categories. Often the minor alerts point to easily fixed oversights, errors and omissions in your CIF or refinement strategy, so attention to these fine details can be worthwhile. In order to resolve some of the more serious problems it may be necessary to carry out additional measurements or structure refinements. However, the purpose of your study may justify the reported deviations and the more serious of these should normally be commented upon in the discussion or experimental section of a paper or in the "special_details" fields of the CIF. checkCIF was carefully designed to identify outliers and unusual parameters, but every test has its limitations and alerts that are not important in a particular case may appear. Conversely, the absence of alerts does not guarantee there are no aspects of the results needing attention. It is up to the individual to critically assess their own results and, if necessary, seek expert advice.

Publication of your CIF in IUCr journals

A basic structural check has been run on your CIF. These basic checks will be run on all CIFs submitted for publication in IUCr journals (*Acta Crystallographica*, *Journal of Applied Crystallography*, *Journal of Synchrotron Radiation*); however, if you intend to submit to *Acta Crystallographica Section C* or *E* or *IUCrData*, you should make sure that full publication checks are run on the final version of your CIF prior to submission.

Publication of your CIF in other journals

Please refer to the *Notes for Authors* of the relevant journal for any special instructions relating to CIF submission.

PLATON version of 06/07/2023; check.def file version of 30/06/2023

Datablock ZrR-1 - ellipsoid plot

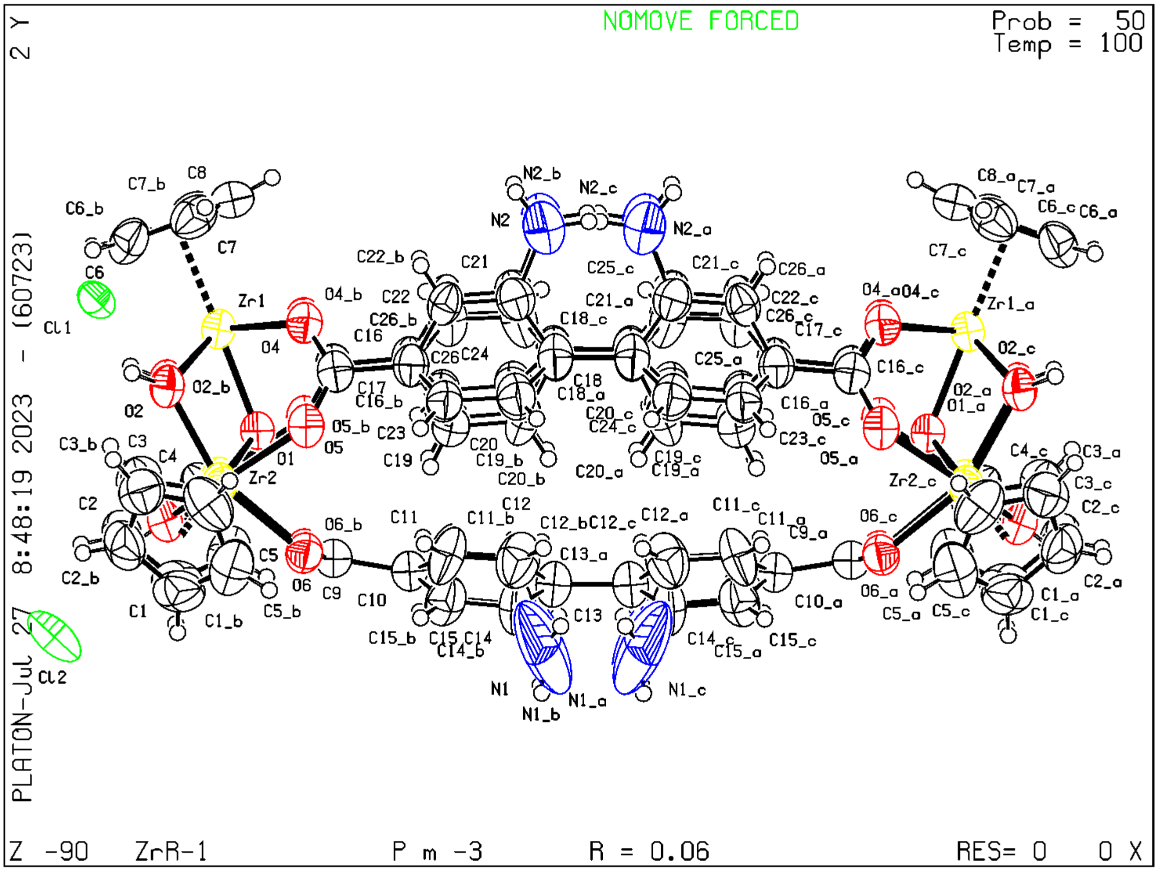

Supplement: Supplementary file 2 — Supporting Information [file ADVS-11-2308445-s002.zip › ZrR-1_checkcif.pdf]
