# Supplementary material for: Hierarchical Self‐Assembly of Capsule‐Shaped Zirconium Coordination Cages with Quaternary Structure
Source: Adv Sci (Weinh). 2024 Jan 16;11(11):2308445. doi: 10.1002/advs.202308445 (PMC10953209; doi:10.1002/advs.202308445)

checkCIF/PLATON report

You have not supplied any structure factors. As a result the full set of tests cannot be run.

THIS REPORT IS FOR GUIDANCE ONLY. IF USED AS PART OF A REVIEW PROCEDURE FOR PUBLICATION, IT SHOULD NOT REPLACE THE EXPERTISE OF AN EXPERIENCED CRYSTALLOGRAPHIC REFEREE.

No syntax errors found. CIF dictionary Interpreting this report

Datablock: ZrR-2

Bond precision:	C-C = 0.0128 A		Wavelength=1.54184
Cell:	a=15.1238(2)	b=15.5214(2)	c=25.2844(3)
	alpha=98.118(1)	beta=95.686(1)	gamma=112.625(1)
Temperature:	150 K		

	Calculated	Reported
Volume	5346.69(13)	5346.69(12)
Space group	P -1	P -1
Hall group	-P 1	-P 1
Moiety formula	C72 H63 N3 O20 Zr6, 3(C3 H7 N O), 2(Cl) [+ solvent]	?
Sum formula	C81 H84 Cl2 N6 O23 Zr6 [+ solvent]	C81 H84 Cl2 N6 O23 Zr6
Mr	2127.77	2127.76
Dx,g cm-3	1.322	1.322
Z	2	2
Mu (mm-1)	5.598	5.598
F000	2140.0	2140.0
F000'	2144.43	
h,k,lmax	19,19,31	19,19,31
Nref	22560	21720
Tmin,Tmax	0.656,0.715	0.850,1.000
Tmin'	0.389	

Correction method= # Reported T Limits: Tmin=0.850 Tmax=1.000
AbsCorr = MULTI-SCAN

Data completeness= 0.963 Theta(max)= 76.803

R(reflections)= 0.0655(17010) wR2(reflections)= 0.1882(21720)

S = 1.031 Npar= 1096

The following ALERTS were generated. Each ALERT has the format
test-name_ALERT_alert-type_alert-level.
Click on the hyperlinks for more details of the test.

Alert level B

```
PLAT097_ALERT_2_B Large Reported Max.  (Positive) Residual Density       4.08 eA-3
PLAT234_ALERT_4_B Large Hirshfeld Difference N6       --C67      .       0.26 Ang.
PLAT420_ALERT_2_B D-H Bond Without Acceptor  O6       --H6O      .     Please Check
```

Alert level C

```
DIFMX02_ALERT_1_C  The maximum difference density is > 0.1*ZMAX*0.75
            The relevant atom site should be identified.
PLAT220_ALERT_2_C NonSolvent   Resd 1  C   Ueq(max)/Ueq(min) Range        3.5 Ratio
PLAT230_ALERT_2_C Hirshfeld Test Diff for    C27      --C28      .        6.1 s.u.
PLAT234_ALERT_4_C Large Hirshfeld Difference N2       --C39      .       0.17 Ang.
PLAT234_ALERT_4_C Large Hirshfeld Difference C24      --C25      .       0.19 Ang.
PLAT234_ALERT_4_C Large Hirshfeld Difference C26      --C30      .       0.23 Ang.
PLAT234_ALERT_4_C Large Hirshfeld Difference C69      --C70      .       0.18 Ang.
PLAT241_ALERT_2_C High   'MainMol' Ueq as Compared to Neighbors of         C5 Check
PLAT241_ALERT_2_C High   'MainMol' Ueq as Compared to Neighbors of         C8 Check
PLAT241_ALERT_2_C High   'MainMol' Ueq as Compared to Neighbors of         C9 Check
PLAT241_ALERT_2_C High   'MainMol' Ueq as Compared to Neighbors of        C15 Check
PLAT241_ALERT_2_C High   'MainMol' Ueq as Compared to Neighbors of        C16 Check
PLAT241_ALERT_2_C High   'MainMol' Ueq as Compared to Neighbors of        C17 Check
PLAT241_ALERT_2_C High   'MainMol' Ueq as Compared to Neighbors of        C21 Check
PLAT241_ALERT_2_C High   'MainMol' Ueq as Compared to Neighbors of        C24 Check
PLAT241_ALERT_2_C High   'MainMol' Ueq as Compared to Neighbors of        C25 Check
PLAT241_ALERT_2_C High   'MainMol' Ueq as Compared to Neighbors of        C26 Check
PLAT241_ALERT_2_C High   'MainMol' Ueq as Compared to Neighbors of        C28 Check
PLAT241_ALERT_2_C High   'MainMol' Ueq as Compared to Neighbors of        C29 Check
PLAT241_ALERT_2_C High   'MainMol' Ueq as Compared to Neighbors of        C30 Check
PLAT241_ALERT_2_C High   'MainMol' Ueq as Compared to Neighbors of        C34 Check
PLAT242_ALERT_2_C Low    'MainMol' Ueq as Compared to Neighbors of        Zr2 Check
PLAT242_ALERT_2_C Low    'MainMol' Ueq as Compared to Neighbors of        Zr4 Check
PLAT242_ALERT_2_C Low    'MainMol' Ueq as Compared to Neighbors of        Zr5 Check
PLAT242_ALERT_2_C Low    'MainMol' Ueq as Compared to Neighbors of        Zr6 Check
PLAT242_ALERT_2_C Low    'MainMol' Ueq as Compared to Neighbors of        C36 Check
PLAT242_ALERT_2_C Low    'MainMol' Ueq as Compared to Neighbors of        C67 Check
PLAT243_ALERT_4_C High   'Solvent' Ueq as Compared to Neighbors of        C79 Check
PLAT244_ALERT_4_C Low    'Solvent' Ueq as Compared to Neighbors of         N7 Check
PLAT244_ALERT_4_C Low    'Solvent' Ueq as Compared to Neighbors of         N8 Check
PLAT244_ALERT_4_C Low    'Solvent' Ueq as Compared to Neighbors of         N9 Check
PLAT244_ALERT_4_C Low    'Solvent' Ueq as Compared to Neighbors of        C76 Check
PLAT250_ALERT_2_C Large U3/U1 Ratio for Average U(i,j) Tensor ....        2.3 Note
PLAT260_ALERT_2_C Large Average Ueq of Residue Including       O22      0.125 Check
PLAT260_ALERT_2_C Large Average Ueq of Residue Including       O23      0.205 Check
PLAT260_ALERT_2_C Large Average Ueq of Residue Including       Cl2      0.121 Check
PLAT342_ALERT_3_C Low Bond Precision on  C-C Bonds ...............    0.01283 Ang.
PLAT420_ALERT_2_C D-H Bond Without Acceptor  N1       --H1N      .     Please Check
```

```
PLAT420_ALERT_2_C D-H Bond Without Acceptor  N1       --H2N      .     Please Check
PLAT420_ALERT_2_C D-H Bond Without Acceptor  N2       --H3N      .     Please Check
PLAT420_ALERT_2_C D-H Bond Without Acceptor  N2       --H4N      .     Please Check
PLAT420_ALERT_2_C D-H Bond Without Acceptor  N4       --H7N      .     Please Check
PLAT420_ALERT_2_C D-H Bond Without Acceptor  N4       --H8N      .     Please Check
PLAT420_ALERT_2_C D-H Bond Without Acceptor  N5       --H9N      .     Please Check
PLAT420_ALERT_2_C D-H Bond Without Acceptor  N6       --H11N     .     Please Check
PLAT420_ALERT_2_C D-H Bond Without Acceptor  N6       --H12N     .     Please Check
```

Alert level G

```
PLAT007_ALERT_5_G Number of Unrefined Donor-H Atoms .............         18 Report
PLAT083_ALERT_2_G SHELXL Second Parameter in WGHT  Unusually Large      17.43 Why ?
PLAT154_ALERT_1_G The s.u.'s on the Cell Angles are Equal ..(Note)      0.001 Degree
PLAT230_ALERT_2_G Hirshfeld Test Diff for    N1       --C36      .        8.5 s.u.
PLAT300_ALERT_4_G Atom Site Occupancy of N1         Constrained at        0.5 Check
PLAT300_ALERT_4_G Atom Site Occupancy of N2         Constrained at        0.5 Check
PLAT300_ALERT_4_G Atom Site Occupancy of N3         Constrained at        0.5 Check
PLAT300_ALERT_4_G Atom Site Occupancy of N4         Constrained at        0.5 Check
PLAT300_ALERT_4_G Atom Site Occupancy of N5         Constrained at        0.5 Check
PLAT300_ALERT_4_G Atom Site Occupancy of N6         Constrained at        0.5 Check
PLAT300_ALERT_4_G Atom Site Occupancy of H1N        Constrained at        0.5 Check
PLAT300_ALERT_4_G Atom Site Occupancy of H2N        Constrained at        0.5 Check
PLAT300_ALERT_4_G Atom Site Occupancy of H3N        Constrained at        0.5 Check
PLAT300_ALERT_4_G Atom Site Occupancy of H4N        Constrained at        0.5 Check
PLAT300_ALERT_4_G Atom Site Occupancy of H5N        Constrained at        0.5 Check
PLAT300_ALERT_4_G Atom Site Occupancy of H6N        Constrained at        0.5 Check
PLAT300_ALERT_4_G Atom Site Occupancy of H7N        Constrained at        0.5 Check
PLAT300_ALERT_4_G Atom Site Occupancy of H8N        Constrained at        0.5 Check
PLAT300_ALERT_4_G Atom Site Occupancy of H9N        Constrained at        0.5 Check
PLAT300_ALERT_4_G Atom Site Occupancy of H10N       Constrained at        0.5 Check
PLAT300_ALERT_4_G Atom Site Occupancy of H11N       Constrained at        0.5 Check
PLAT300_ALERT_4_G Atom Site Occupancy of H12N       Constrained at        0.5 Check
PLAT300_ALERT_4_G Atom Site Occupancy of H36        Constrained at        0.5 Check
PLAT300_ALERT_4_G Atom Site Occupancy of H39        Constrained at        0.5 Check
PLAT300_ALERT_4_G Atom Site Occupancy of H50        Constrained at        0.5 Check
PLAT300_ALERT_4_G Atom Site Occupancy of H53        Constrained at        0.5 Check
PLAT300_ALERT_4_G Atom Site Occupancy of H64        Constrained at        0.5 Check
PLAT300_ALERT_4_G Atom Site Occupancy of H67        Constrained at        0.5 Check
PLAT301_ALERT_3_G Main Residue  Disorder .............(Resd  1  )         3% Note
PLAT414_ALERT_2_G Short Intra D-H..H-X       H1N      ..H43      .       1.95 Ang.
                                                     x,y,z  =     1_555 Check
PLAT414_ALERT_2_G Short Intra D-H..H-X       H3N      ..H34      .       1.92 Ang.
                                                     x,y,z  =     1_555 Check
PLAT414_ALERT_2_G Short Intra D-H..H-X       H6N      ..H57      .       2.05 Ang.
                                                     x,y,z  =     1_555 Check
PLAT414_ALERT_2_G Short Intra D-H..H-X       H8N      ..H48      .       2.06 Ang.
                                                     x,y,z  =     1_555 Check
PLAT414_ALERT_2_G Short Intra D-H..H-X       H11N     ..H62      .       1.94 Ang.
                                                     x,y,z  =     1_555 Check
PLAT415_ALERT_2_G Short Inter D-H..H-X       H7       ..H12N     .       2.13 Ang.
                                                  x,-1+y,z  =     1_545 Check
PLAT415_ALERT_2_G Short Inter D-H..H-X       H10N     ..H77C     .       1.92 Ang.
                                                  -1+x,y,z  =     1_455 Check
PLAT415_ALERT_2_G Short Inter D-H..H-X       H11N     ..H80B     .       2.08 Ang.
                                                     x,y,z  =     1_555 Check
PLAT606_ALERT_4_G Solvent Accessible VOID(S) in Structure .......          ! Info
```

```
PLAT790_ALERT_4_G Centre of Gravity not Within Unit Cell: Resd.  #          4 Note
              C3 H7 N O
PLAT790_ALERT_4_G Centre of Gravity not Within Unit Cell: Resd.  #          6 Note
              Cl
PLAT794_ALERT_5_G Tentative Bond Valency for Zr1       (IV)      .       4.13 Info
PLAT794_ALERT_5_G Tentative Bond Valency for Zr2       (IV)      .       4.15 Info
PLAT794_ALERT_5_G Tentative Bond Valency for Zr3       (IV)      .       4.13 Info
PLAT794_ALERT_5_G Tentative Bond Valency for Zr4       (IV)      .       4.19 Info
PLAT794_ALERT_5_G Tentative Bond Valency for Zr5       (IV)      .       4.18 Info
PLAT794_ALERT_5_G Tentative Bond Valency for Zr6       (IV)      .       4.16 Info
PLAT869_ALERT_4_G ALERTS Related to the Use of SQUEEZE  Suppressed          ! Info
PLAT883_ALERT_1_G No Info/Value for _atom_sites_solution_primary .     Please Do !
PLAT933_ALERT_2_G Number of HKL-OMIT Records in Embedded .res File          1 Note
PLAT941_ALERT_3_G Average HKL Measurement Multiplicity ..........         3.4 Low
```

---

0 **ALERT level A** = Most likely a serious problem - resolve or explain
3 **ALERT level B** = A potentially serious problem, consider carefully
46 **ALERT level C** = Check. Ensure it is not caused by an omission or oversight
50 **ALERT level G** = General information/check it is not something unexpected

3 ALERT type 1 CIF construction/syntax error, inconsistent or missing data
48 ALERT type 2 Indicator that the structure model may be wrong or deficient
3 ALERT type 3 Indicator that the structure quality may be low
38 ALERT type 4 Improvement, methodology, query or suggestion
7 ALERT type 5 Informative message, check

It is advisable to attempt to resolve as many as possible of the alerts in all categories. Often the minor alerts point to easily fixed oversights, errors and omissions in your CIF or refinement strategy, so attention to these fine details can be worthwhile. In order to resolve some of the more serious problems it may be necessary to carry out additional measurements or structure refinements. However, the purpose of your study may justify the reported deviations and the more serious of these should normally be commented upon in the discussion or experimental section of a paper or in the "special_details" fields of the CIF. checkCIF was carefully designed to identify outliers and unusual parameters, but every test has its limitations and alerts that are not important in a particular case may appear. Conversely, the absence of alerts does not guarantee there are no aspects of the results needing attention. It is up to the individual to critically assess their own results and, if necessary, seek expert advice.

Publication of your CIF in IUCr journals

A basic structural check has been run on your CIF. These basic checks will be run on all CIFs submitted for publication in IUCr journals (*Acta Crystallographica*, *Journal of Applied Crystallography*, *Journal of Synchrotron Radiation*); however, if you intend to submit to *Acta Crystallographica Section C* or *E* or *IUCrData*, you should make sure that full publication checks are run on the final version of your CIF prior to submission.

Publication of your CIF in other journals

Please refer to the *Notes for Authors* of the relevant journal for any special instructions relating to CIF submission.

PLATON version of 06/07/2023; check.def file version of 30/06/2023

Datablock ZrR-2 - ellipsoid plot

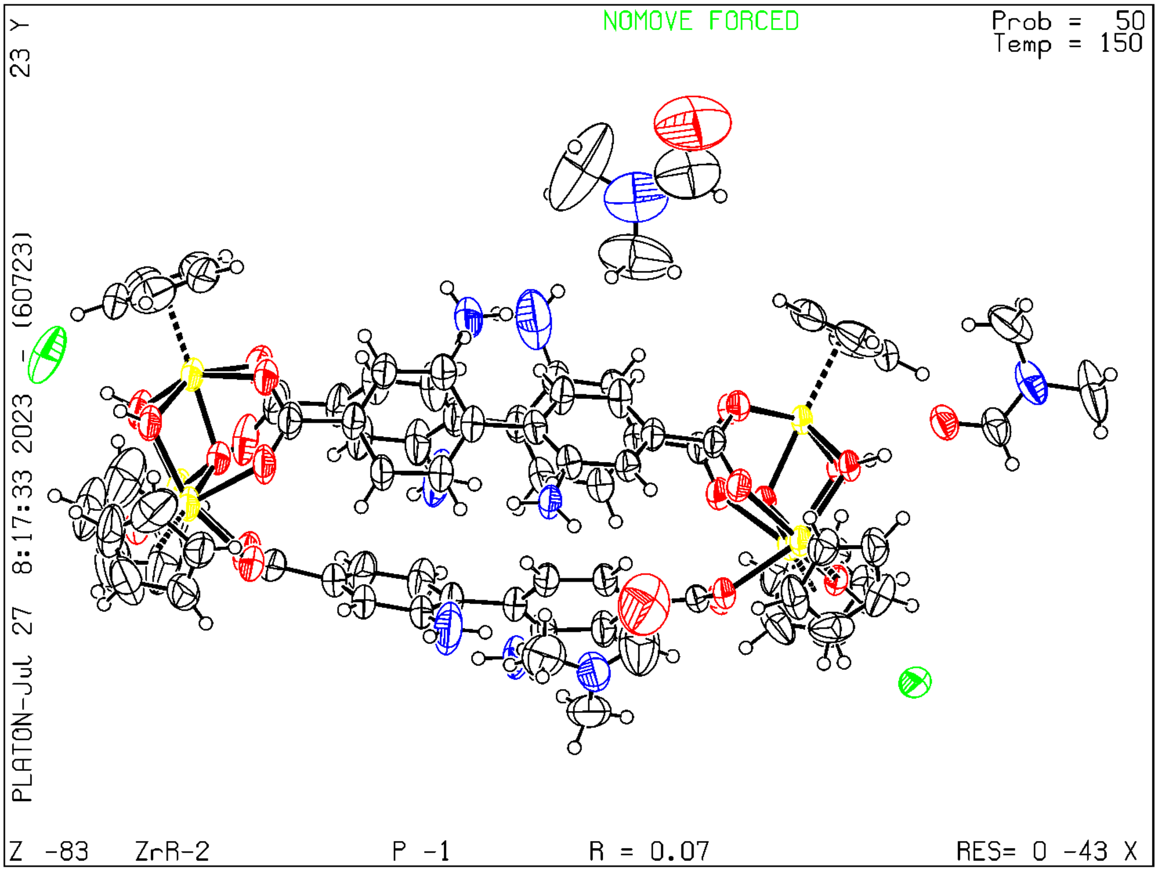

Supplement: Supplementary file 2 — Supporting Information [file ADVS-11-2308445-s002.zip › ZrR-2_checkcif.pdf]
